# Function and Interaction of the Coupled Genes Responsible for *Pik-h* Encoded Rice Blast Resistance

**DOI:** 10.1371/journal.pone.0098067

**Published:** 2014-06-04

**Authors:** Chun Zhai, Yu Zhang, Nan Yao, Fei Lin, Zhe Liu, Zhongqiu Dong, Ling Wang, Qinghua Pan

**Affiliations:** 1 National Key Laboratory for Conservation and Utilization of Subtropical Agro-bioresources, South China Agricultural University, Guangzhou, China; 2 Guangdong Provincial Key Laboratory for Microbe Signals and Crop Disease Control, South China Agricultural University, Guangzhou, China; 3 National Key Laboratory of Biocontrol, College of Life Sciences, Sun Yat-sen University, Guangzhou, China; Universidad Miguel Hernández de Elche, Spain

## Abstract

*Pik-h*, an allele of *Pik*, confers resistance against the rice blast pathogen *Magnaporthe oryzae*. Its positional cloning has shown that it comprises a pair of NBS-LRR genes, *Pikh-1* and *Pikh-2*. While *Pikh-1* appears to be constitutively transcribed, the transcript abundance of *Pikh-2* responds to pathogen challenge. The Pikh-1 CC (coiled coil) domain interacts directly with both AvrPik-h and Pikh-2. Transient expression assays demonstrated that Pikh-2 mediates the initiation of the host defence response. Nucleocytoplasmic partitioning of both Pikh-1 and Pikh-2 is required for their functionalities. In a proposed mechanistic model of *Pik-h* resistance, it is suggested that Pikh-1 acts as an adaptor between AvrPik-h and Pikh-2, while Pikh-2 transduces the signal to trigger *Pik-h-*specific resistance.

## Introduction

The surveillance system evolved by plants to detect and defend against microbial attack requires a capacity to recognize a plethora of pathogens. The result of detection and successful defense is often the hypersensitive response (HR; [1]), which is triggered when a pathogen's avirulence (*Avr*) gene is recognized by a matching host resistance (*R*) gene. The genetic relationship between the *R* and *Avr* genes was elegantly interpreted by Flor in 1971, and modeled in his “gene-for-gene” hypothesis [2]. However, genome-wide analyses have increasingly revealed that the host *R* gene repertoires are far smaller than what would be needed to respond specifically to every pathogen that it may encounter [3], [4]. This fact implies that individual *R* genes must have the capacity to recognize more than one pathogen race and even family [5], [6]. Although some broad-spectrum recognition mechanisms not involving *R* genes have been described, multiple recognition specificity is considered necessary for the perception by *R* genes of a wide variety of pathogens [6]–[8]. The co-evolution of host and pathogen drives the mutation of Avr proteins to alter their recognition properties, with allelic diversity at *R* genes representing the response [9], [10].

Numerous *R* genes have been isolated in recent years. Most have proven to encode a large cytoplasmic protein comprising either an N-terminal coiled coil (CC) or a Toll-interleukin receptor (TIR) domain, a central nucleotide-binding site (NBS) domain, and a C-terminal leucine-rich repeat region (LRR) [11]. The NBS domain shares a degree of sequence similarity with mammalian apoptosis-inducing effectors, and its function has therefore been considered to be a switch regulating R protein activity. The LRR region is generally thought to represent the major determinant of specificity [12]. The TIR/CC domain was initially proposed to function in downstream signalling, as shown in flax L10 and *Arabidopsis thaliana* RPP1 [13], [14]. However, accumulating evidence also supported the role of this domain in effector recognition, including the cases of the *A. thaliana* RPS5, the tobacco N proteins [15], [16], and the potato RB proteins [8]. Therefore, the TIR/CC domain has become the subject of intense interest for its dual functions in “gene-for-gene” interactions. Recently, the importance of this domain for R protein function was further highlighted in two studies, which demonstrated that TIR/CC domain-dependent dimerization is required for downstream immune signaling [17], [18].

Plant R proteins have been found to locate in diverse sub-cellular compartments. In the past few years, a number of studies have situated the nucleus in the center of attention for disease resistance activation. For instance, upon elicitor induction, several R proteins, including the tobacco N, the barley MLA10, and the *A. thaliana* RPS4 and SNC1 proteins, were found to accumulate in the nucleus, and this nuclear accumulation is required for their proper functioning [19]–[23]. However, two recent studies demonstrated that the nucleocytoplasmic potato Rx protein requires both nuclear and cytoplasmic pools for its activation [24], [25]. Apart from nucleocytoplasmic distribution, R proteins may also be found in the plasma membrane, as is the case with *A. thaliana* RPM1 [26]. Through nuclear exclusion and direct membrane tethering, Gao *et al*. showed that activation and signalling of RPM1 occurs at the plasma membrane and initiates a cytosolic pathway [26]. Therefore, plant R proteins can function in specific cellular compartments depending on the protein.

Some examples have emerged where resistance against a single pathogen requires not just one, but a pair of NBS-LRR genes [27]. The earliest example is related to resistance against *Peronospora parasitica* in *A. thaliana* [28], but subsequently, the same scenario has been established for a viral resistance in tobacco [29], leaf rust resistance in wheat [30], blast resistance in rice [31]–[35], and the resistance against three distinct pathogens in *A. thaliana* [4]. As yet it is still unclear how such coupled genes can act co-operatively to establish effector recognition, but detailed examination of the *Pik* locus in rice has shown that resistance does indeed require functional versions of both genes to be present.

Here we report the function of and the nature of the interaction between the coupled genes responsible for *Pik-h* encoded rice blast resistance. Genetic analysis has established that a gene-for-gene interaction exists between various *AvrPik* and *Pik* alleles [34]–[37]. Here, we have elaborated a working model for the AvrPik-h and Pik-h interaction, in which one of the *R* gene products (Pikh-1) acts as an adaptor between AvrPik-h and the other *R* gene product (Pikh-2), while Pikh-2 transduces the signal to trigger the *Pik-h-*specific resistance.

## Results

### Identification and Validation of Candidates for *Pik-h*

The *Pik-h* donor cultivar (cv.) K3 was crossed with the blast susceptible cv. As20-1, and *Pik-h* resistance in the resulting F_2_ population segregated in a monogenic fashion [resistant/susceptible: 484/150 (χ_c_^2^ = 0.54; *P*>0.40)]. Linkage analysis placed *Pik-h* within a 237 kb interval flanked by markers K37 (proximal) and K28 (distal), a location which is identical to that of both *Pik* and *Pik-p*, in which two haplotypes K and N have been shown to differ by a large insertion/deletion (Table S1; Figure S1; [34], [35]).

Since cvs K3 (*Pik-h* carrier), K60 (*Pik-p*) and Kusabue (*Pik*) all possess the K haplotype [35], the *Pikp-1/Pik-1* pair (for simplicity, the K1 gene) and the *Pikp-2/Pik-2* pair (K2 gene) were considered to be the most likely candidates for *Pik-h* (Figure S1). A gain-of-function test based on the introduction into susceptible cvs of one of three distinct constructs- the first harboring *Pikh-1* alone, the second *Pikh-2* alone and the third both *Pikh-1* and *Pikh-2* (referred to as *Pikh-12*)- was performed. Transgenic plants carrying *Pikh-1* or *Pikh-2* constructs alone did not gain a complementary phenotype, while those carrying the *Pikh-12* construct in susceptible backgrounds did (Table 1; Figure S2), indicating that the expression of *Pik-h* resistance requires two independent genetic determinants. Loss-of-function tests of *Pikh-1* and *Pikh-2* independently further confirmed that both genes were involved in *Pikh-*mediated blast resistance (Table 1; Figure S2).

**Table 1:** Characterization of the coupled genes for *Pik-h* via gain and loss of function approaches.

	Recipient	Inoculation	Reaction of T_0_ Plants a					Success
Candidate/construct	Cultivar	Isolate	R	MR	MS	S	Total	ratio (%) b
Gain of function								
*Pikh-1*	Q1063	CHL346	0	0	1	149	150	0
*Pikh-2*	Q1063	CHL346	0	0	0	82	82	0
*Pikh-12*	Kuyuku 131	CHL42	7	3	1	8	19	42.1
*Pikh-12*	K60	CHL42	82	14	14	4	114	84.2
Loss of function c								
*Pikh-1* RNAi (KP3i1)	K3	CHL346	21	0	4	17	42	50.0
*Pikh-2* RNAi (KP4i)	K3	CHL346	5	0	0	20	25	80.0

a T_0_ plants derived from each construct were inoculated with the *Pikh*-avirulent isolates, CHL346 or CHL42. R, resistant; MR, moderate resistant; MS, moderate susceptible; S, susceptible.

b The success ratios for gain- and loss-of-function complementation tests were calculated as (R+MR)/(R+MR+MS+S), and (MS+S)/(R+MR+MS+S), respectively.

c The *Pik* allele-specific RNAi constructs, KP3 RNAi1 and KP4 RNAi, which correspond to *Pikp-1* and *Pikp-2*, respectively [40], were transformed into the *Pik-h* carrier cv K3.

Sequence data from this article can be found in the GenBank/EMBL databases under accession numbers HQ662330 (*Pik-h*).

### Transcription Profiles of *Pikh-1* and *Pikh-2*

**Figure 1:**
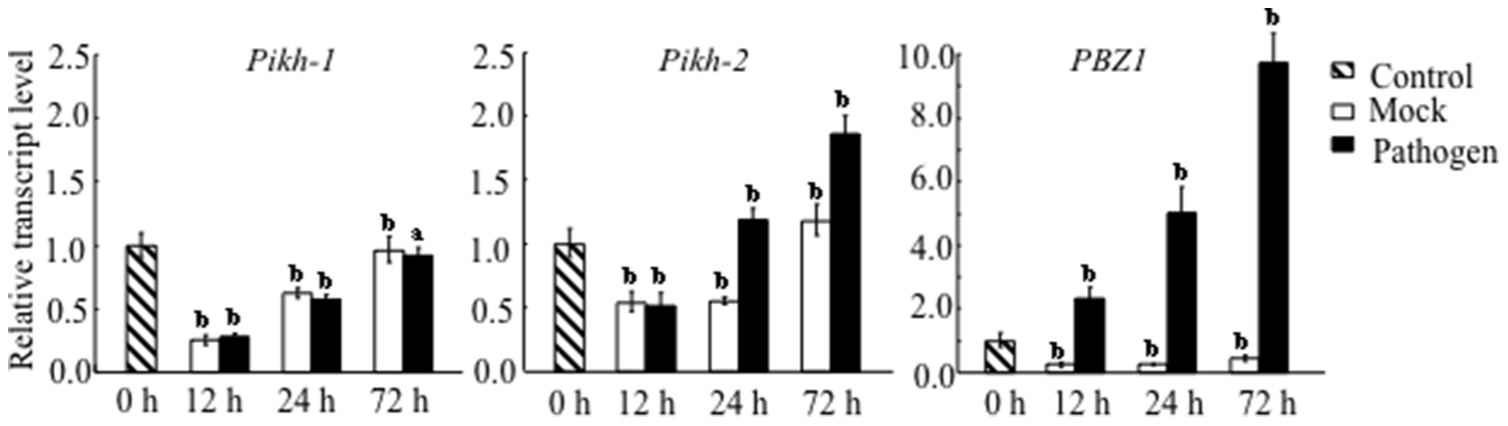
Transcription profiles of the coupled genes *Pikh-1* and *Pikh-2*, as assayed by qRT-PCR. Data represent mean ± standard deviation (n = 3). Similar results were obtained from two independent experiments. Lower case letter (a, b) used to indicate whether the treatment and control means differed at *P*<0.01 or *P*<0.05 level, respectively. The pathogen-inducible gene *PBZ1* [46] was used as a reference.

The transcript abundance of *Pikh-1* in plants infected by an avirulent race of rice blast fell over the first 12 h following inoculation, but then increased steadily to its constitutive level by 72 h. Since the same profile was observed in the mock-inoculated control (Figure 1), this response was most likely associated with the inoculation procedure itself rather than to an interaction with the pathogen. However, the transcription of *Pikh-2* rose after 12 h, reaching about two fold of the constitutive level by 72 h after inoculation. Given the different expression pattern of *Pikh-1* and *Pikh-2*, it is tempting to speculate that the coupled genes were not co-regulated upon the pathogen challenge.

### Interactions among Pikh-1, Pikh-2 and AvrPik-h *in Vitro*

**Figure 2:**
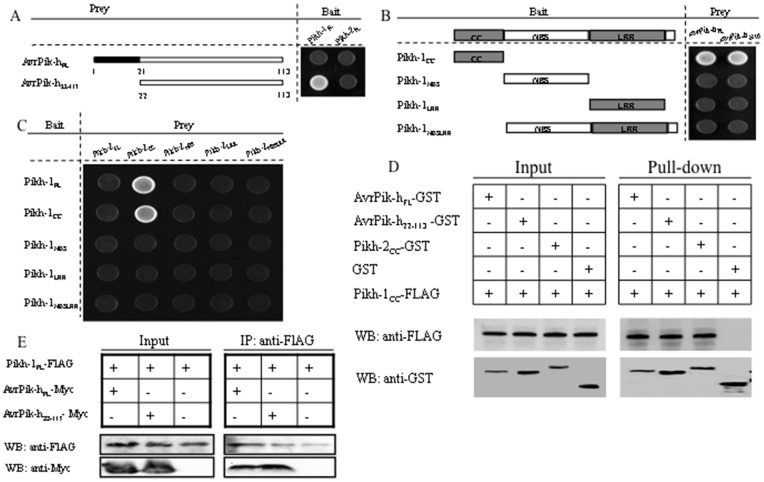
Interactions among Pikh-1, Pikh-2 and AvrPik-h proteins as detected by the yeast two hybrid, pull-down, and co-immunoprecipitation systems. (**A**) Pikh-1_FL_ and Pikh-2_FL_ *vs* full length or truncated AvrPik-h proteins. (**B**) Defining the Pikh-1 domain interacting with AvrPik-h_FL_ and AvrPik-h_22–113_. (**C**) Full length and truncated Pikh-1 *vs* Pikh-2. (**D**) Pikh-1_CC_ *vs* AvrPik-h_FL_, AvrPik-h_22–113_ and Pikh-2_CC_ using the pull-down assay. (**E**) Pikh-1_FL_ *vs* AvrPik-h_FL_and AvrPik-h_22–113_ using the co-immunoprecipitation system. Western blots employed anti-FLAG (Pikh-1_CC_ and Pikh-1_FL_), anti-GST (AvrPik-h_FL_, AvrPik-h_22–113_ and Pikh-2_CC_), and anti-Myc (AvrPik-h_FL_ and AvrPik-h_22–113_) antibodies.

Yeast two-hybrid assays demonstrated that the full-length Pikh-1 (Pikh-1_FL_) protein, but not Pikh-2, interacted with the cognate mature protein, AvrPik-h_22–113_ (a signal peptide truncated form of AvrPik-h, identical to AvrPik-D [37]) (Figure 2A). The implication was that Pikh-2 is unlikely to be receptor for effector recognition. When a series of truncated Pikh-1 proteins was tested for interaction with both the full-length and the truncated forms of AvrPik-h, it was apparent that the CC domain (Pikh-1_CC_) was both necessary and sufficient for the interaction to occur (Figure 2B). Interactions were also detected between Pikh-2_CC_ and both Pikh-1_FL_ and Pikh-1_CC_ (Figure 2C). These interactions were verified by the pull-down and co-immnoprecipitation assays (Figures 2D and 2E). The expression of all bait and prey proteins was confirmed by Western blot analysis (Figure S4A).

### Interactions among Pikh-1, Pikh-2 and AvrPik-h *in vivo*

When GFP-fused proteins consisting of either the full-length or isolated domains of the three genes were separately expressed in rice protoplasts, distinct distributions of GFP signal were observed. The Pikh-1_FL_-GFP construct demonstrated a balanced distribution between cytoplasm and nucleus, whereas the Pikh-1_CC_-GFP exclusively accumulated in nucleus. A higher level of fluorescence signal of the Pikh-1_NBS_-GFP was detected in the nucleus than in cytoplasm, while the Pikh-1_LRR_-GFP was excluded from the nucleus and distributed throughout the cytoplasm (Figure S5A). In contrast, both Pikh-2_FL_-GFP and Pikh-2_CC_-GFP accumulated in both the nucleus and the cytoplasm, and both Pikh-2_NBS_-GFP and Pikh-2_LRR_-GFP exclusively, in the nucleus (Figure S5B). Additionally, both AvrPik-h_FL_-GFP and AvrPik-_h22–113_-GFP distributed between cytoplasm and nucleus (Figure S5C). As expected, the nuclear localization signal (NLS) fused versions of both Pikh-1_FL_-GFP and Pikh-2_FL_-GFP accumulated in the nucleus, while the nuclear export signal (NES) carrying versions of both the full-length and CC domain of the coupled genes excluded from the nucleus (Figure S5D).

**Figure 3:**
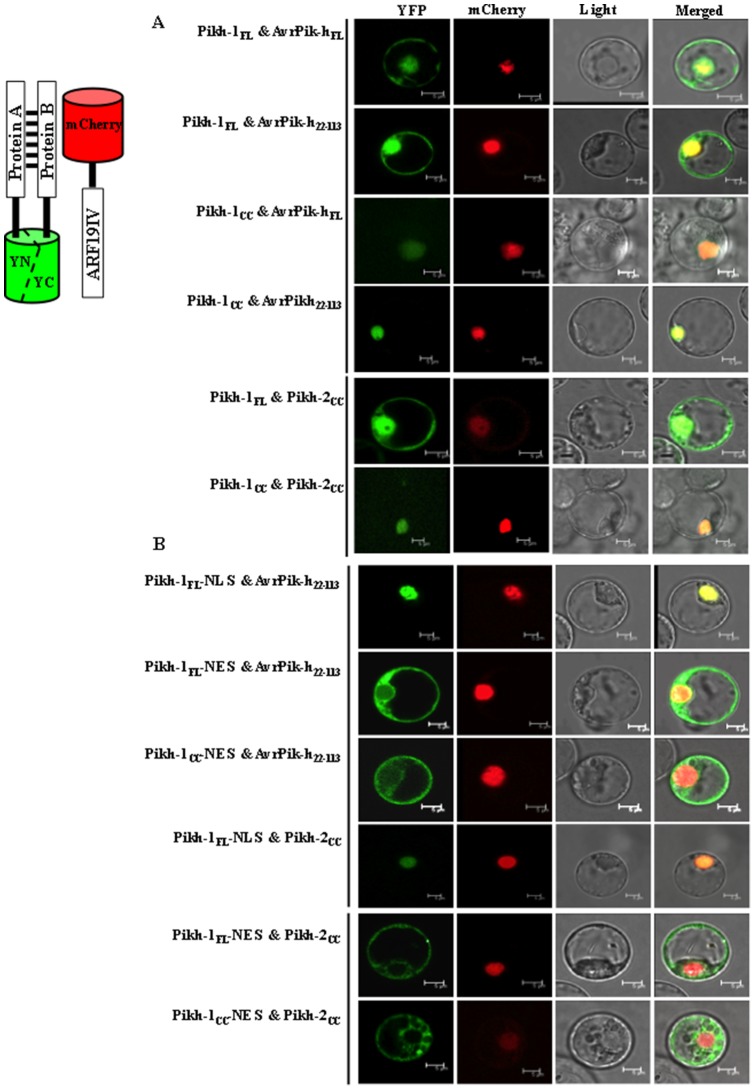
Interactions among Pikh-1, Pikh-2, and AvrPik-h proteins as detected by bimolecular fluorescence complementation (BiFC) assay. The images shown are representative of at least three independent experiments. (**A**) Pikh-1_FL_ complexed with AvrPik-h_FL_, AvrPik-h_22–113_, and Pikh-2_CC_ in both the nucleus and the cytoplasm, while Pikh-1_CC_ produces such interactions only in the nucleus. (**B**) The targeting signals NLS and NES redirected interactions to, respectively, the nucleus and the cytoplasm. ARF19IV-mCherry, nuclear marker.

The positive interactions observed *in vitro* were further confirmed by the bimolecular fluorescence complementation (BiFC) assay in a rice protoplast system. The localization and distribution of all the combinations of the triple proteins was stable, but not dynamic between cellular organs. That is, The Pikh-1_FL_ protein formed a complex with either AvrPik-h_FL_ or AvrPik-h_22–113_ in both the cytoplasm and the nucleus, but the Pikh-1_CC_ altered this nucleocytoplasmic partitioning to nucleus, only (Figure 3A). The Pikh-1_FL_ protein also formed a complex with Pikh-2cc in both the cytoplasm and nucleus, and once again its replacement with Pikh-1_CC_ also disrupted the nucleocytoplasmic distribution of the Pikh-1 interaction with Pikh-2 (Figure 3A). When the NES versions of both Pikh-1_FL_ and Pikh-1_CC_ were co-expressed with the AvrPik-h_22–113_ or Pikh-2 _CC_, fluorescence was detected only in the cytoplasm, while the NLS versions were detected exclusively in the nucleus (Figure 3). As the negative controls, neither Pikh-1_FL_ nor AvrPik-h_FL_ gave BiFC with Pikh-2_FL_ (Figure S6, upper panel). In addition, co-expression of various versions of Pikh-1, Pikh-2, and AvrPik-h proteins with the non-allelic blast *R* gene protein, Pib, did not produce any fluorescence (Figure S4B; and Figure S6, lower panel). Moreover, a nucleocytoplasmic fractionation-based Western blot assay was applied to assure the localizations of interactions of Pikh-1_FL_ and Pikh-1_CC_ with AvrPik-h_22–113_. As expected, the former combination was detected in both nuclear and the nuclei-depleted fractions, and the latter one was detected only in the nuclei fraction (Figure S7). Together, these indicated that the fluorescence detected was due to a specific association of the proteins related.

### Functionalities upon interactions among Pikh-1, Pikh-2 and AvrPik-h

**Figure 4:**
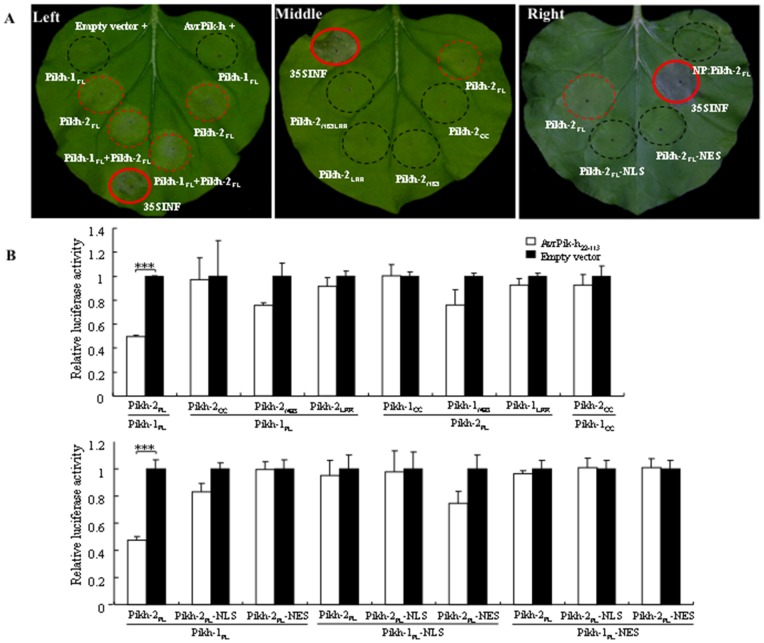
Functionality upon interactions among Pikh-1, Pikh-2, and AvrPik-h proteins. (**A**) Transient expression in *N*. *benthamiana*. Pikh-2 on its own triggered an HR-like response (left). The Pik-h domains required for the HR (middle). The response induced by various versions of Pikh-2 (right). The NES or NLS redirected distributions of Pikh-2 resulted in the loss of its functionality. NP: Native promoter. The p35S-INF construct was used as positive control. (**B**) Transient assay of HR in cv. Nipponbare protoplasts using firefly luciferase as a reporter. The *R* gene constructs were driven by their NP, and *Avr* by the maize ubiquitin promoter. The reduction in luminescence was compared with the control, where an empty vector, instead of AvrPik-h_22–113_, was uesd for protoplast transformation. Each assay consisted of three technical and three biological replicates. ***P<0.001. Co-expression of the full length Pikh-1 and Pikh-2 was necessary to confer *AvrPik-h*-dependent HR, and the NES or NLS redirected subcellular distributions of Pikh-1 and Pikh-2 resulted in loss of functionality.

The molecular basis for Pik-h activation was investigated via an *Agrobacterium*-mediated transient expression system in *Nicotiana benthamiana*, in which an HR-like response was induced by the co-expression of various combinations of the Pik-h and AvrPik-h proteins. All combinations involving Pikh-2_FL_ induced an HR-like response, demonstrating that it could be auto-activated under the control of the CaMV 35S promoter (Figure 4A, left panel). The analysis of truncated Pikh-2 derivatives showed that only the Pikh-2_FL_ protein was able to induce HR (Figure 4A, middle panel). Since the Pikh-2_FL_ is deposited in both the nucleus and cytoplasm (Figure S5B), a pair of constructs of Pikh-2_FL_ fused to either NLS or NES was generated to test whether the subcellular distribution of Pikh-2 is of any importance for its autoactivity. This experiment showed that the restriction of Pikh-2 within either the nucleus or cytoplasm blocked its HR-triggering activity (Figure 4A, right panel; Figure S5D), which implied that both nuclear and cytoplasmic pools of Pikh-2 are needed for normal function. The expression of the Pikh-2_FL_ protein driven by its native promoter (NP) likewise induced no HR (Figure 4A, right panel). Immunoblot analysis revealed that except for the Pikh-2 protein driven by NP, the proteins all accumulated at a comparable level (Figure S4C). Thus, the HR induction shown by the Pikh-2 protein driven by the CaMV 35S promoter may have derived from its higher level of accumulation.

Since auto-activation has been observed in heterologous plant expression systems, a firefly luciferase reporter gene-based HR-like cell death assay was performed in rice protoplasts to investigate how Pik-h might function. As the full-length AvrPik-h protein displayed auto-activation, while the signal peptide truncated construct ubi::AvrPik-h_22–113_ did not (Figure S8), the latter was challenged by both Pikh-1 and Pikh-2 expressed by their NP (Figure 4B, upper panel). Only the combination of AvrPik-h_22–113_ and Pikh-1_FL_ and Pikh-2_FL_ resulted in a significant reduction in luciferase activity. In contrast, the combination among AvrPik-h_22–113_ and Pikh-1_FL_ protein with any Pikh-2 truncated protein, or AvrPik-h_22–113_ and Pikh-1 truncated protein with the Pikh-2_FL_ protein did not induce significant HR-like cell death in rice protoplasts. Interestingly, combination of AvrPik-h_22–113_, and the CC domains of both Pikh-1 and Pikh-2 did not cause significant cell death, indicating that the parsimonious regions involved in the protein-protein interactions aforementioned were insufficient to trigger the effector-dependent HR.

To further determine whether the nucleocytoplasmic partitioning was required for the *Pikh*-mediated resistance, both Pikh-1 and Pikh-2 were fused either to NLS or NES and expressed under the control of their NP in rice protoplasts. Combinations involving AvrPik-h_22–113_ and the NLS or NES versions of Pikh-1_FL_ with any of the Pikh-2 versions induced no HR-like cell death and *vice versa* (Figure 4B, lower panel), even though the NLS- or NES-tagged proteins were as abundant as the wild types (Figure S4D). Hence, it is tempting to speculate that the balanced distribution of Pikh-1 and Pikh-2 in both nucleus and cytoplasm is essential for the *Pikh*-mediated resistance.

## Discussion

### Positional divergence of the coupled genes for the *Pik* alleles

Although the majority of R proteins share a tripartite structure consisting of a CC/TIR, an NBS domain and an LRR region, the role of each of these components is no longer as clear cut as was once believed. The NBS domain was initially thought to regulate the activation of the R protein [38], but the observation that the expression of just the NBS domain of the potato Rx protein on its own is sufficient to trigger a constitutive defence response has led to the modified theory that it can also serve as an interaction platform for studying downstream signalling components [11], [39]. The LRR region was generally held to be responsible for Avr perception, as it has clearly been subjected to strong diversifying selection. Compelling evidence for this role has been provided by both *in vitro* or *in vivo* assays of the rice Pita and the *A. thaliana* RPP1 proteins [14], [40], where in both cases the LRR region was necessary and sufficient for the R/Avr interaction. The supposed function of the CC/TIR domain was associated with downstream signalling [13], [14], but it has become clear that it is also involved with pathogen recognition [15], [16]. According to the previous studies, all of the CC/TIR domains which mediate the R/Avr interaction were assembled in those R proteins being interacted with Avr proteins, indirectly, compared with the LRR domain in those R proteins interacting with Avr proteins, directly [15], [15,41]. However, it is the CC domain of Pikh-1 which is necessary and sufficient for direct binding with AvrPik-h (Figures 2B and 2D), consistent with the observation that the variable amino acids in Pikh-1 were found mostly within its CC domain (Figure S3A). The present results supply a clear example for the mediation of pathogen perception by direct interaction with an N-terminal domain. The Pikh-1 CC domain was also responsible for the interaction between Pikh-1 and Pikh-2 (Figures 2C and 2D), implying that it may act as a molecular bridge relaying a signal from AvrPik-h to Pikh-2. It has been reported that self-association of the L6 TIR domain, which could provide a scaffold for downstream signaling protein binding, is a requirement for immune activation [17]. Similarly, the crystal structure of the MLA CC domain has revealed that the CC dimer serves as a minimal functional module in cell death initiation [18]. The CC domain-dependent homodimerization of MLA has been suggested to attract particular WRKY hetero- or homo-oligomers for the purpose of downstream signaling [18]. Whether the heterodimer of the CC/TIR domain is therefore also a critical player in downstream signaling remains an open question. However, the co-expression of the CC domains of both Pikh-1 and Pikh-2 was insufficient to induce HR (Figure 4B), suggesting that the association of the two CC domains may be related to the formation of AvrPikh-Pikh1-Pikh2 recognition complexes rather than specifically related to a downstream signaling event.

The sub-cellular localization of R proteins is relevant to their activity in resistance signalling. The tobacco *R* gene product N, which confers resistance against tobacco mosaic virus (TMV), is found in both the cytoplasm and the nucleus. During TMV infection, the shuttling of p50-activated N from the cytoplasm to the nucleus appears to be required for an effective defence response [19]. As a second example, the activity of the *A. thaliana* SNC1 and RPS4 have also been associated with their nuclear accumulation [21], [22]. Strikingly, a recent study has confirmed that the enhanced defense responses mounted under conditions of ABA deficiency and high temperature is dependent on the nuclear localization of SNC1 and RPS4 [23]. In contrast, the regulation of the potato CC-NBS-LRR Rx protein (which confers resistance against potato virus X) requires its nucleocytoplasmic distribution [24], [25], which is brought about by its CC and LRR domains and facilitated by an accessory protein. While the nuclear Rx protein may play a role in transcriptional reprogramming, the cytoplasmic form mounts a defence process against the virus [24], [25]. With respect to the *Pik-h* product, the Pikh-1 CC domain disrupted its balanced nucleocytoplasmic partitioning through its interaction with both AvrPik-h and Pikh-2 (Figure 3A). When GFP fusion proteins comprising either the full-length Pikh-1 or specific Pikh-1 domains were expressed in rice protoplasts, it was only the former which retained a balanced distribution between the cytoplasm and the nucleus (Figure S5A). Thus, similar to the situation with Rx, the Pikh-1 CC and LRR domains may well have contrasting roles in determining the cellular distribution of the full-length Pikh-1 protein. Given that the full-length Pikh-1 protein was required for HR induction (Figure 4B), and that it interacted with AvrPik-h or Pikh-2 in both nucleus and cytoplasm (Figure 3A), the assumption is that *Pikh-*mediated resistance relies on its balanced cellular distribution. Indeed, the enforced restriction of Pikh-1 to either the nucleus or the cytoplasm blocked the AvrPikh-dependent cell death (Figure 4B). The full-length and CC domain of Pikh-2 accumulated mostly, and the NBS and LRR domains exclusively, in the nucleus (Figure S5B). An enhanced nuclear or cytoplasmic accumulation of Pikh-2 also compromised its HR activity (Figures 4A and 4B). The inference is that in the expression of *Pik-h* resistance, Pikh-2 may interact with Pikh-1 in the nucleus to direct transcriptional reprogramming, and in the cytoplasm to recruit certain critical downstream signaling machineries.

### Functional divergence of the coupled *Pik* genes

Characterizing the function of each constituent of a coupled gene system like Pik-h would represent a major step towards understanding the mechanisms of *R* gene systems. The transcription profiles of *Pikh-1* and *Pikh-2* clearly differ from one another, with the former being constitutively expressed and the latter responding to pathogen challenge (Figure 1). A possible scenario is that the constitutive expression of *Pikh-1* is required for elicitor recognition, whereas the inducible expression of *Pikh-2* reflects its participation in downstream signalling following pathogen infection. The coupled genes (*Pi5-1* and *Pi5-2*) responsible for *Pi5-*encoded blast resistance have similar transcription profiles with that of *Pikh-1* and *Pikh-2*, respectively [32], which may represent a mechanism shared by a number of the dual *R-*genes systems. *Pik-1* sequences were more divergent than those of *Pik-2* (Figures S3A and S3B), which suggests that positive selection has been operating more heavily on the former alleles. The induction of HR-like cell death in *N*. *benthamiana* required the presence of the full length Pikh-2 protein (Figure 4A), and this was also needed to induce HR-like cell death in rice protoplasts in the presence of both Pikh-1 and AvrPik-h (Figure 4B). The over-expression of NRG1, a signalling component of the defence response, causes ectopic cell death in the absence of its partner, N [29]. In line with this observation, Pikh-2 (rather than Pikh-1) appears to be the protein responsible for the activation of downstream signalling. Finally, Pikh-2 was found to interact directly with Pikh-1, but not with AvrPik-h (Figure 2).

**Figure 5:**
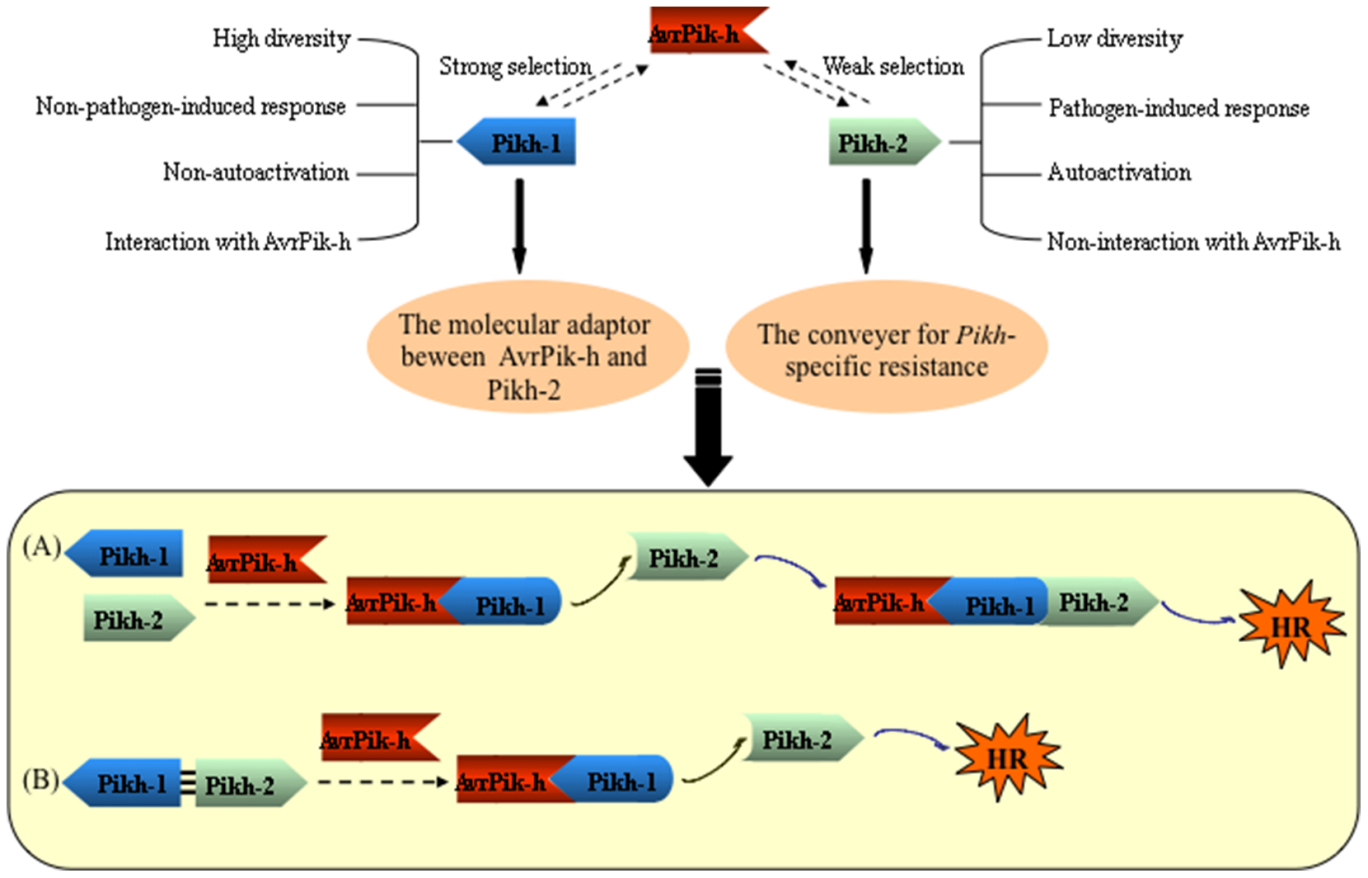
A proposed model for the function of the coupled *Pik*-h genes. (**A**) Pikh-1 perceives the Avr signal and relays it to Pikh-2, whereupon Pikh-2 defence response. (**B**) Pikh-1 is constitutively associated with Pikh-2. The recognition of AvrPik-h destabilizes this association, thereby lifting the constraints on the conformation of Pikh-2, and restoring its signalling potential.

Two leading models have been elaborated to explain how a single plant R protein responds to its cognate pathogen effector. In the ligand-receptor model, recognition occurs as a result of a direct physical association between the Avr and the R proteins [9], [40], [42], [43]. In the second model, the R protein recognizes the matching Avr in a more mechanistically complex fashion, involving an accessory protein [44]. The Avr effector modifies the accessory protein in a way which the R protein perceives as a signal, and this then triggers the defence response. The association of the R protein with the accessory protein can be either constitutive [15], or can occur only after the Avr-induced modification of the accessory protein [16]. When two *R* genes are required for resistance, the likely situation is that the more parsimonious strategy is adopted, in which one gene is involved in elicitor recognition via direct interaction, and the other acts as a downstream component. In the case of the *Pik-h* resistance, Pikh-1 appears to represent a molecular adaptor between AvrPik-h and Pikh-2, while Pikh-2 is a genuine conveyer for the *Pik-h-*specific resistance. Two possible mechanisms underlying Pik-h activation are illustrated in Figure 5. In the absence of the Avr effector, both Pikh-1 and Pikh-2 are maintained in an inactive state. Once the presence of AvrPik-h has been detected, Pikh-1 adopts a signalling-competent conformation, which relays a signal to Pikh-2, inducing a conformational change. The consequent activation of Pikh-2 allows it to recruit specific transcription factors in the nucleus and the necessary downstream signaling proteins in the cytoplasm, thereby triggering the defence response (Figure 5A). Alternatively, Pikh-1 is constitutively associated with Pikh-2 *in planta*. Recognition of AvrPik-h de-stabilizes this association, which releases Pikh-2 from its structural constraints. The unconstrained conformation of Pikh-2 unlocks its signalling potential, resulting in the initiation of the necessary transcriptional reprogramming and the recruitment of the critical downstream signaling machinery (Figure 5B; [28]). The identification of the signal transduction pathway and the definition of its functional structures have become the focus of our continuing research into this important crop plant *R* gene.

While this paper was under preparation, Kanzaki et al. [37], who reported that the binding specificity between Pik-1 and AvrPik allelic proteins correlates with the recognition (race) specificity between *Pik* and *AvrPik* alleles. We, however, cannot verify the relationships with the Pik-1 allelic proteins against the AvrPik allelic proteins. As shown in Figure S9, physical interactions were also observed between all the five Pik-1 alleles (Piks-1, Pikp-1, Pik-1, Pikm-1, and Pikh-1, considering either the full-length protein, the CC domain, and the RAXT1 motif) and three AvrPik-h alleles, AvrPik-D (AvrPik-h), AvrPik-E, and AvrPik-A [37], as well as Pikh-2_CC_ (the Pik-2 alleles all share an identical CC domain; Figure S3B). This suggests that the Pik-1 alleles show no differential binding specificity toward either AvrPik-h or any of the Pikh-2 alleles. The non-differential binding between Pikh-1 and AvrPik-h allelic proteins may be due to the presence of the RATX1 motifs in the Pik-1 CC domains (Figure S3A), which have been recognized as an Avr recognition motif [6]. The Pik-1 protein, in short, interacts with not only AvrPik, but also AvrPia proteins [6]. Similarly, there was also no binding specificity between 17 CC domains present in the potato RB protein derived from ten wild species (amino acid sequence similarities between 65.9% and 98.2%) and the relevant *P. infestans* effector family IPI-O1 (avirulent) and IPI-O4 (virulent) [8]. Such results raise the question of how recognition (race) specificity relates to binding specificity.

## Materials and Methods

### High-resolution genetic mapping

A mapping population consists of 634 F_2_ progeny of the cross between the *Pik-h* donor cv. K3 and the blast susceptible cv. As20-1. The rough location of the *R* gene was initially identified using bulk segregant analysis [45], and then mapped more precisely using a set of genomic position-ready markers previously used for characterizing the *Pik-p*/*Pik* region [34], [35].

### Complementation analysis

The cloning strategy applied for *Pik-p* and *Pik* [34], [35] was adopted to form constructs for the candidates *Pikh-1* and *Pikh-2*, along with a fused candidate *Pikh-12*. All the constructs were validated by sequencing before being introduced as transgenes into the blast susceptible recipient cvs Q1063, Kuyuku131 and K60. To test for loss-of-function, two *Pik* allele-specific RNAi constructs (KP3 RNAi1 and KP4 RNAi), which target, respectively, *Pikp-1* and *Pikp-2* [34], were transformed into the *Pik-h* carrier cv. K3. Transgenic progeny were tested for reaction to blast infection with isolate CHL346 (virulent to cv. Q1063, and avirulent to cv. K3) or CHL42 (virulent to cvs Kuyuku 131 and K60, and avirulent to cv. K3), and their transgenic status was confirmed using a PCR-based assay directed against the respective construct, as described previously [35].

### Transcription analysis

Seedlings of cv. K3 grown to the three leaf stage in a pathogen-free room at 25°C were inoculated with either blast isolate CHL346 or water (mock inoculation, also called pathogen infection control), and held in the dark at 25°C under 100% relative humidity for 20 h. Since the *Pik* alleles were sensitive to environmental conditions, such as temperature, light, and humidity, seedlings being grown in a pathogen-free tissue room were used as an environmental control (34). The *Pik-h* transcription profile was obtained using quantitative RT-PCR (qRT-PCR), as described previously [35]. In brief, total RNA was treated with RNase-free DNase I (Promega, Madison, WI, USA), and reverse transcribed by M-MLV (Promega). The quality of RNA was assessed by measuring absorbance at 230, 260 and 280 nm, respectively, with a NanoDrop spectrophotometer (Thermo Scientific, Wilmington, DE, USA). Integrity of the RNA was verified by gel electrophoresis on an ethidium bromide-stained 1% agarose-TBE gel and denaturing agarose-MOPS gel. Only the RNA samples with A260/A280 ratios between 1.8 and 2.1 and A260/A230 ratios higher than 2.0, as well as the both 28S and 18S ribosomal RNA bands with a density ratio about 2∶1 were subjected to qRT-PCR analysis. qRT-PCR was performed using SYBR Premix Ex Taq (TaKaRa, Dalian, China), on a Bio-Rad CFX96 Real-Time PCR Detection System device (Bio-Rad, Hercules, CA, USA). The rice housekeeping gene encoding *Actin*, which was used for the expression analyses of *Pik-m* (31) and *Pik-p* (34), was used as an endogenous control to normalize the RNA expression in each qRT-PCR. The *Pik* allele-specific primer sets RRT5 and RRT17 (31), respectively, were employed to detect *Pikh-1* and *Pikh-2* expression. Primer efficiency was verified using standard curves generated by plotting the amount of the mixed cDNA against the threshold cycle (*Ct*) value for a series of dilutions (six orders of magnitude). Expression of genes was calculated via the 2^−ΔΔCt^ method. Two biological and three technical replicates were included. The pathogen-inducible gene *PBZ1* [31], [46] was used as a reference. Statistical comparisons were conducted with ANOVA test using SPSS v16.0 software (http://www-01.ibm.com/software/analytics/spss/).

### Yeast two hybrid analysis

The full-length and truncated cDNAs corresponding to *Pikh-1*, *Pikh-2* and *AvrPik-h* (AB498875.1; [47]) were cloned in frame with the GAL4 DNA binding domain (BD) of the bait vector pGBKT7, and the GAL4 activation domain (AD) of the prey vector pGADT7, respectively. The joint transformation of a BD and an AD construct into Y2HGold yeast cells was performed using the Matchmaker Gold yeast two hybrid system (Clontech, CA. USA), following the manufacturer's instructions. Yeast transformants were grown on minimal media lacking leucine and tryptophan to select for the presence of both constructs, and potential protein-protein interactions were confirmed by growing the cells in selective minimal media lacking leucine, tryptophan, histidine and adenine. Standard positive (pGBKT7-53 and pGADT7-T) and negative (pGBKT7-Lam and pGADT7-T) controls were included in each experiment. In addition, BD constructs of the five *Pik-1* alleles were also generated and subjected to test for the specificity of interaction as described above. Yeast proteins tested were extracted by the trichloro-acetic acid method, separated by SDS-PAGE, and transferred to nitrocellulose membranes (Bio-Rad) by electroblotting in a Bio-Rad Mini Trans-Blot apparatus. Membranes were blocked with 3% w/v skimmed milk, and probed with an anti-Myc or an anti-HA monoclonal antibody (Sigma, MO, USA) followed by detection with anti-rabbit IgG HRP conjugates (Bio-Rad). Labeling was detected with the ECL plus Western blotting detection system (GE Healthcare, UK).

### *In vitro* GST pull-down assay

The CC domain of *Pikh-1* was inserted into the pF3A WG vector fused to a FLAG tag construct (Promega), while the *Pikh-2_CC_*, and intact and truncated fragments of *AvrPik-h* were inserted into the pGEX-6P-1 vector to form a GST-fusion construct (GE Healthcare). The GST-fused proteins expressed in *E. coli* strain BL21 were used to pull down the FLAG-fused proteins synthesized in a wheat germ-derived *in vitro* transcription and translation system (Promega) using a MagneGST pull-down system (Promega), following the manufacturer's protocol. The proteins eluted from the beads were separated through a 12% SDS-PAGE gel and transferred to a nitrocellulose membrane (Bio-Rad). Protein blots were blocked with 3% w/v skimmed milk and then probed with either an anti-GST or an anti-FLAG monoclonal antibody (Sigma). Either goat anti-mouse (Sigma) or anti-rabbit IgG HRP conjugates (Bio-Rad) was used as the secondary antibody for subsequent detection via enhanced chemiluminescence (GE Healthcare). As an additional recognition specificity assay, two sets of the five *Pik* alleles, three versions of *AvrPik-h* alleles, as well as the Pikh-2_CC_ (the Pik-2 alleles share an identical CC domain) were tested by the above procedure.

### Co-immunoprecipitation assay

For the coimmunoprecipitation and immunoblot assay, *Pikh-1*, and *AvrPik-h* and *AvrPik-h_22–113_* fragments were ligated into the binary vectors, pCXSN-FLAG and pCXSN-Myc [48], respectively. Cultures of *Agrobacterium tumefaciens* strain GV3101 carrying one of these constructs were incubated at 28°C overnight in LB medium containing the appropriate antibiotic selective agent. When the culture OD_600_ had reached ∼1.0, the cells were pelleted and re-suspended in 10 mM MgCl_2_, 10 mM MES, 200 µM acetosyringone, the OD_600_ was adjusted to 1.5, and the cells then held at room temperature for 3 h. The cultures were combined in an equimolar mixture, and infiltrated into the leaves of three-week old *N*. *benthamiana* plants using a 1 ml needle-less syringe. Leaf samples were collected two days after infiltration, and then homogenized with lysis buffer (50 mM Tris-HCl, pH 7.4, 100 mM KCl, 2.5 mM MgCl_2_, 0.1% v/v NP-40, 5 mM DTT, and a protease inhibitor cocktail [Roche, IN, USA]), and centrifuged at 15,000 rpm for 30 min at 4°C. Immunoprecipitation was performed using a Co-immunoprecipitation Kit (Pierce, IL, USA), following the manufacturer's protocol, and immunoblots were conducted as described above.

### Sub-cellular localization and the BiFC assay

To generate the fluorescent constructs, PCR amplified fragments of the target genes were ligated in frame to the C terminus of the eGFP coding region of pUC18 and expressed under the control of the CaMV 35S promoter. To establish BiFC plasmid constructs, the *Pikh-1* full length cDNA or its CC domain sequence were fused with the C terminal fragment of the gene encoding yellow fluorescent protein (YFP) in the pUC-pSPYCE vector [49], while the *Pikh-2* CC and the intact and various truncated forms of *AvrPik-h* were fused with the N terminal fragment of YFP in the same vector. For mislocalization analysis, either the NLS or NES segment were amplified, respectively, from the pMD20T-NLS (the vector harboring an annealed oligo SV40 NLS; [50]) and pMD20T-NES (the vector harboring an annealed oligo PK1 NES; [51]) plasmids with forward and reverse primers containing appropriate restriction sites, and inserted into the GFP-fused or BiFC constructs. Since a monopartite NLS in the DNA-binding domains of the rice auxin response factor ARF19 is responsible for its nuclear localization [52], a truncated element containing the monopartite NLS (ARF19IV) fused to mCherry was prepared in a pMD20T vector (TaKaRa) to generate a *mCherry::ARF19IV* construct, which was then used to assess nuclear localization. The resulting constructs were used in transient assays based on the polyethylene glycol-mediated transfection of rice protoplasts isolated from ten day old cv. Nipponbare seedlings [53], [54]. After a 30 h incubation at 22°C, the protoplasts were examined by confocal microscopy (Leica TCS SP5), and representative images were shown. Each experiment was repeated at least three times with similar results using independent samples, and at least 30 protoplasts were observed in each independent sample. Nuclear and cytosolic fractions in rice protoplasts expressing various combinations of the BiFC constructs were separated by using a Sub-cellular Fractionation Kit (BestBio, Shanghai, China), following the manufacturer's protocol, and immunoblots were conducted as described above.

### Transient expression in *N. benthamiana*

*Pikh-1*, *Pikh-2* and *AvrPik-h* fragments were ligated into the binary vectors, pCXSN-FLAG, pCXSN-HA and pCXSN-Myc [48], respectively. To create the redirected Pikh-2 versions, the NLS and NES segments were introduced into the Pikh-2 construct as described above. In addition, the HA-Pikh-2 segment under the control of its native promoter was cloned into the pCAMBIA1300 vector. Cultures of *A. tumefaciens* strain GV3101 carrying one of these constructs were infiltrated into the leaves of five-week old *N*. *benthamiana* plants according to the procedure above-mentioned. The appearance of HR was monitored three days after infiltration. The p35S-INF1 [55] construct was used as a positive control in each experiment.

### Transient expression in rice protoplasts

*Pikh-1* and *Pikh-2* fragments under the control of their respective native promoters, as well as *AvrPik-h* fragments under the control of the maize ubiquitin promoter, were each inserted into the pMD20T vector. The FLAG-NLS, FLAG-NES, HA-NLS and HA-NES fragments were amplified, respectively, from the constructs created for the transient expression assays, and inserted to the Pikh-1 or Pikh-2 constructs. The resulting constructs, along with a reporter plasmid construct which contained the firefly luciferase gene, were co-transfected into rice protoplasts using a polyethylene glycol-based method [53], [54]. An estimate of the concentration of protoplasts in each transfection reaction was determined using BCA protein assay kit (Pierce). Protoplasts were harvested by centrifugation 40 h after incubation at 22°C, and a luciferase assay conducted using a Luciferase Assay System (Promega). Luciferase activity was set in relation to cell concentration. Each assay consisted of three technical and three biological replicates. All statistical analyses were conducted using SPSS v16.0 software (http://www-01.ibm.com/software/analytics/spss/). For protein expression analysis, PEG-mediated transfections and total protein extraction were performed as described previously [54].

## Supporting Information

Figure S1 **The genomic location of** ***Pik-h*** **.**
(TIFF)

Figure S2 **Molecular characterization of** ***Pik-h*** **related transgenic progeny.**
(TIFF)

Figure S3 **Alignment of predicted polypeptide sequences encoded by the** ***Pik*** **paired genes. (A) Pik-1/Pikm-1/Piks-1/Pikp-1/Pikh-1; (B) Pik-2/Pikm-2/Piks-2/Pikp-2/Pikh-2.**
(PPT)

Figure S4 **Confirmation of protein expression by Western blot analysis.** (**A**) Yeast two-hybrid assay; (**B**) Negative control for BiFC assay; (**C**) *N. benthamiana-*based transient assay; (**D**) Rice protoplast-based luciferase assay.
(TIFF)

Figure S5 **Sub-cellular localization of the full-length and truncated versions of three proteins involved in** ***Pik-h*** **-mediated resistance.** (**A**) Pikh-1 and a GFP alone control; (**B**) Pikh-2 and a GFP alone control; (**C**) AvrPik-h; (**D**) Mislocation of Pikh-1 and Pikh-2 by using NLS and NES.
(PPT)

Figure S6 **Negative controls for BiFC assay.**
(TIFF)

Figure S7 **Cell fractionation-based Western blot assay.**
(TIFF)

Figure S8 **Assessment of the autoactivity of AvrPik-h_FL_, AvrPik-h_FL_, Pikh-1_FL_ and Pikh-2_FL_ using a rice protoplast-based luciferase assay.**
(TIFF)

Figure S9 **The relationship between recognition (race) and binding specificity of the** ***Pik*** **and** ***AvrPik*** **alleles.** (**A–D**) The binding specificity of the five Pik allelic proteins: full-length (FL), CC domain, and RAXT1 motif. Three AvrPik alleles (AvrPik-D, -E, and –A; [37]), and Pikh-2_CC_ (FL level only) were selected for binding specificity test via yeast two hybrid system (Clontech, CA). (**E**) Race specificity of the five *Pik* alleles. Five monogenic lines for each of the *Pik* alleles tested with the three *AvrPik* alleles via general pathotype test.
(TIFF)

Table S1 **Primers, markers and constructs used in the study.**
(PDF)
