# Supplementary material for: Function and Interaction of the Coupled Genes Responsible for Pik-h Encoded Rice Blast Resistance
Source: PLoS One. 2014 Jun 4;9(6):e98067. doi: 10.1371/journal.pone.0098067 (PMC4045721; doi:10.1371/journal.pone.0098067)
Supplement: Figure S3 — Alignment of predicted polypeptide sequences encoded by the Pik paired genes. (A) Pik-1/Pikm-1/Piks-1/Pikp-1/Pikh-1; (B) Pik-2/Pikm-2/Piks-2/Pikp-2/Pikh-2. (PPT) [file pone.0098067.s003.ppt]

## Slide 1
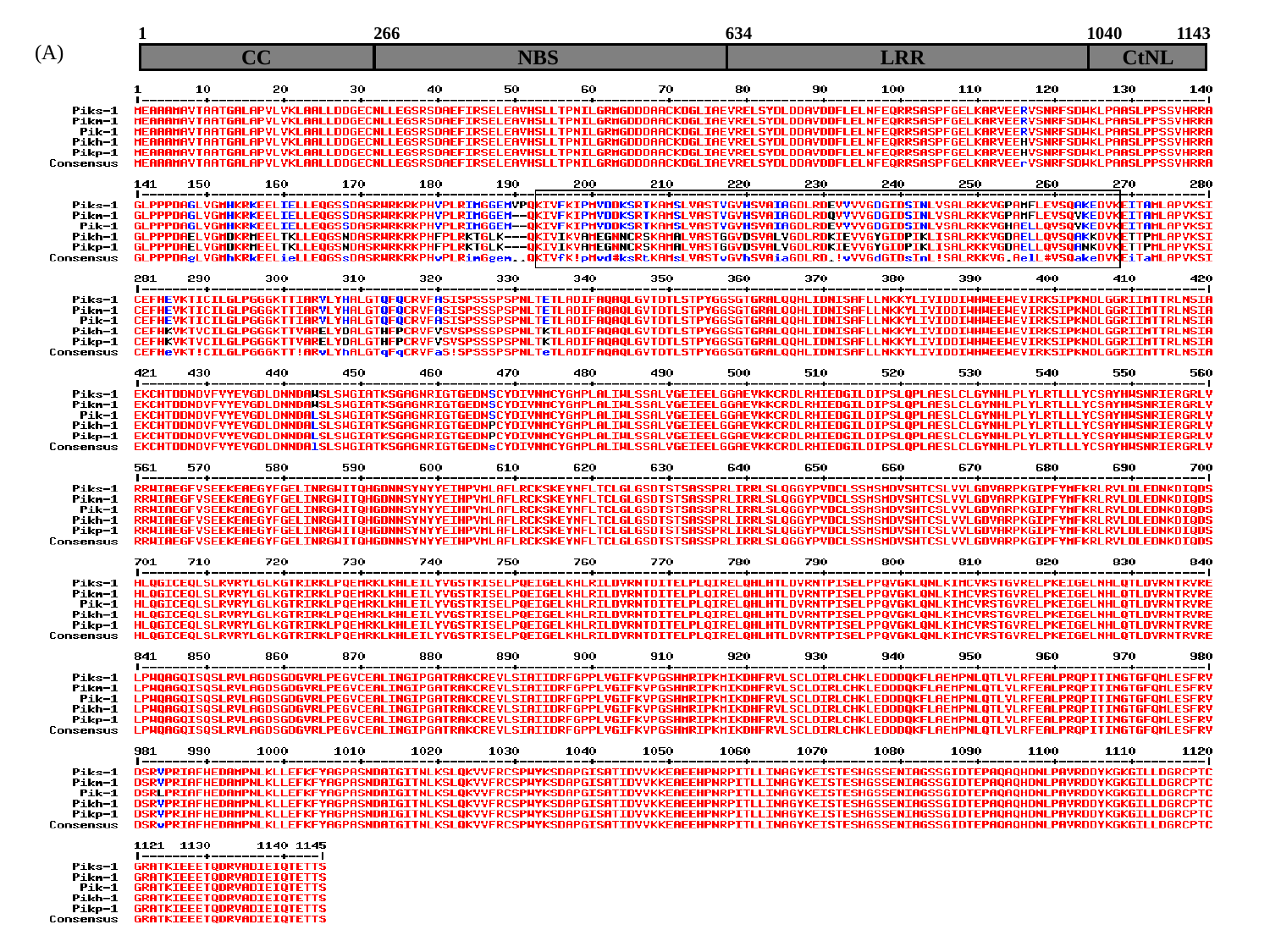

266
634
1143
1
1040
NBS
LRR
CC
CtNL
(A)

## Slide 2
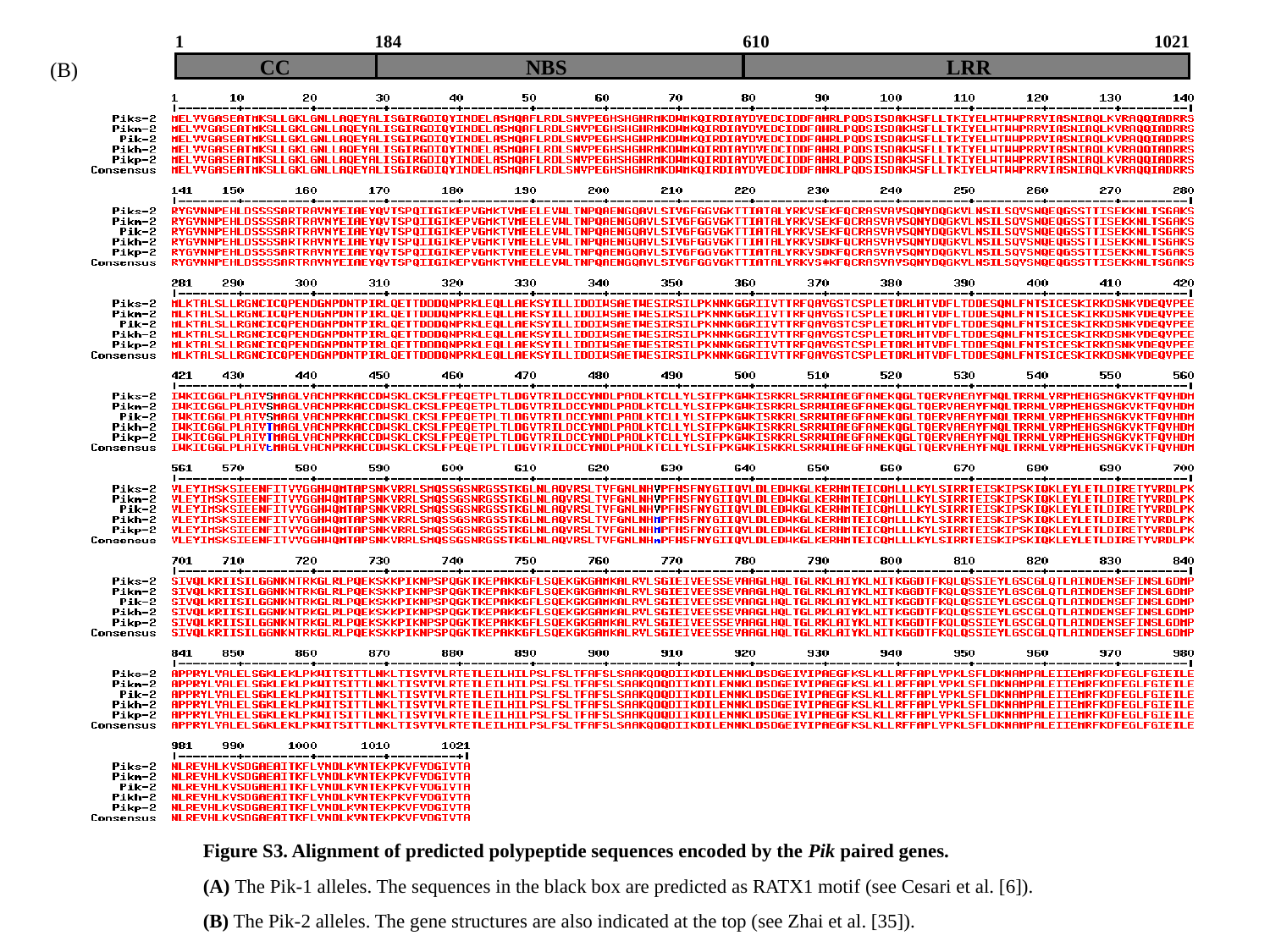

184
610
1
1021
CC
NBS
LRR
(B)
Figure S3. Alignment of predicted polypeptide sequences encoded by the Pik paired genes.
(A) The Pik-1 alleles. The sequences in the black box are predicted as RATX1 motif (see Cesari et al. [6]).
(B) The Pik-2 alleles. The gene structures are also indicated at the top (see Zhai et al. [35]).
