# Supplementary figures and images for: Function and Interaction of the Coupled Genes Responsible for Pik-h Encoded Rice Blast Resistance
Source: PLoS One. 2014 Jun 4;9(6):e98067. doi: 10.1371/journal.pone.0098067 (PMC4045721; doi:10.1371/journal.pone.0098067)

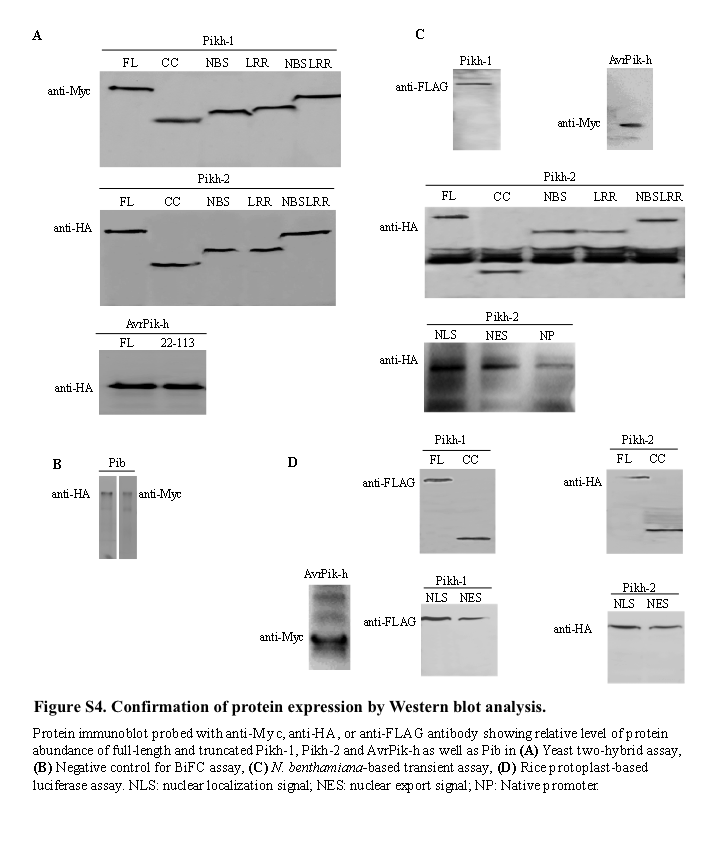

Supplement: Figure S4 — Confirmation of protein expression by Western blot analysis. (A) Yeast two-hybrid assay; (B) Negative control for BiFC assay; (C) N. benthamiana-based transient assay; (D) Rice protoplast-based luciferase assay. (TIFF) [file pone.0098067.s004.tif]

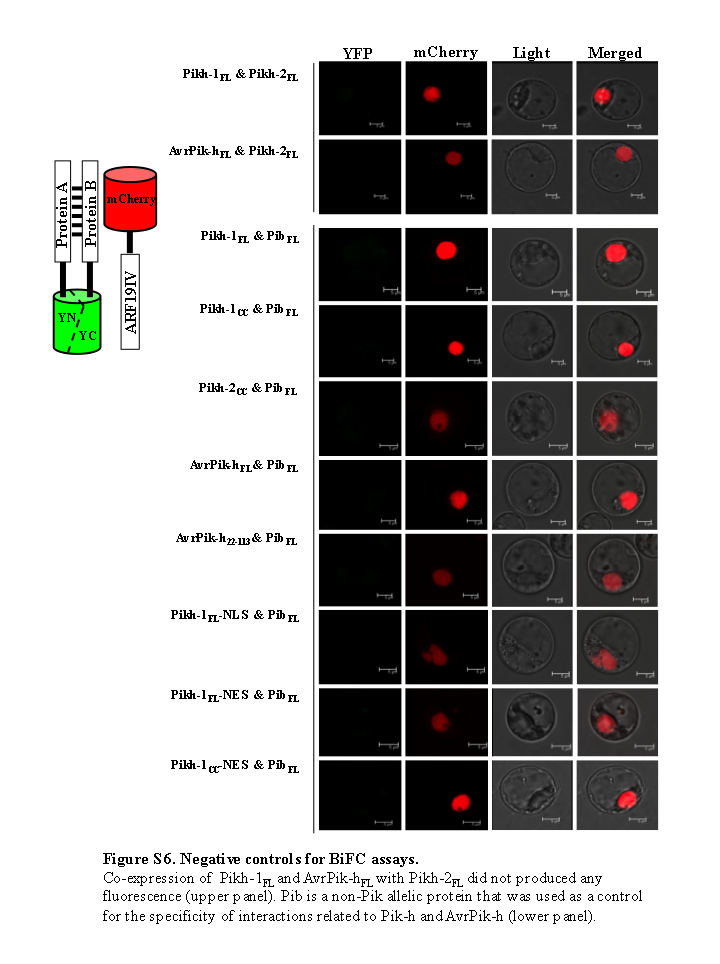

Supplement: Figure S6 — Negative controls for BiFC assay. (TIFF) [file pone.0098067.s006.tif]
