# Supplementary material for: Function and Interaction of the Coupled Genes Responsible for Pik-h Encoded Rice Blast Resistance
Source: PLoS One. 2014 Jun 4;9(6):e98067. doi: 10.1371/journal.pone.0098067 (PMC4045721; doi:10.1371/journal.pone.0098067)
Supplement: Figure S5 — Sub-cellular localization of the full-length and truncated versions of three proteins involved in Pik-h -mediated resistance. (A) Pikh-1 and a GFP alone control; (B) Pikh-2 and a GFP alone control; (C) AvrPik-h; (D) Mislocation of Pikh-1 and Pikh-2 by using NLS and NES. (PPT) [file pone.0098067.s005.ppt]

## Slide 1
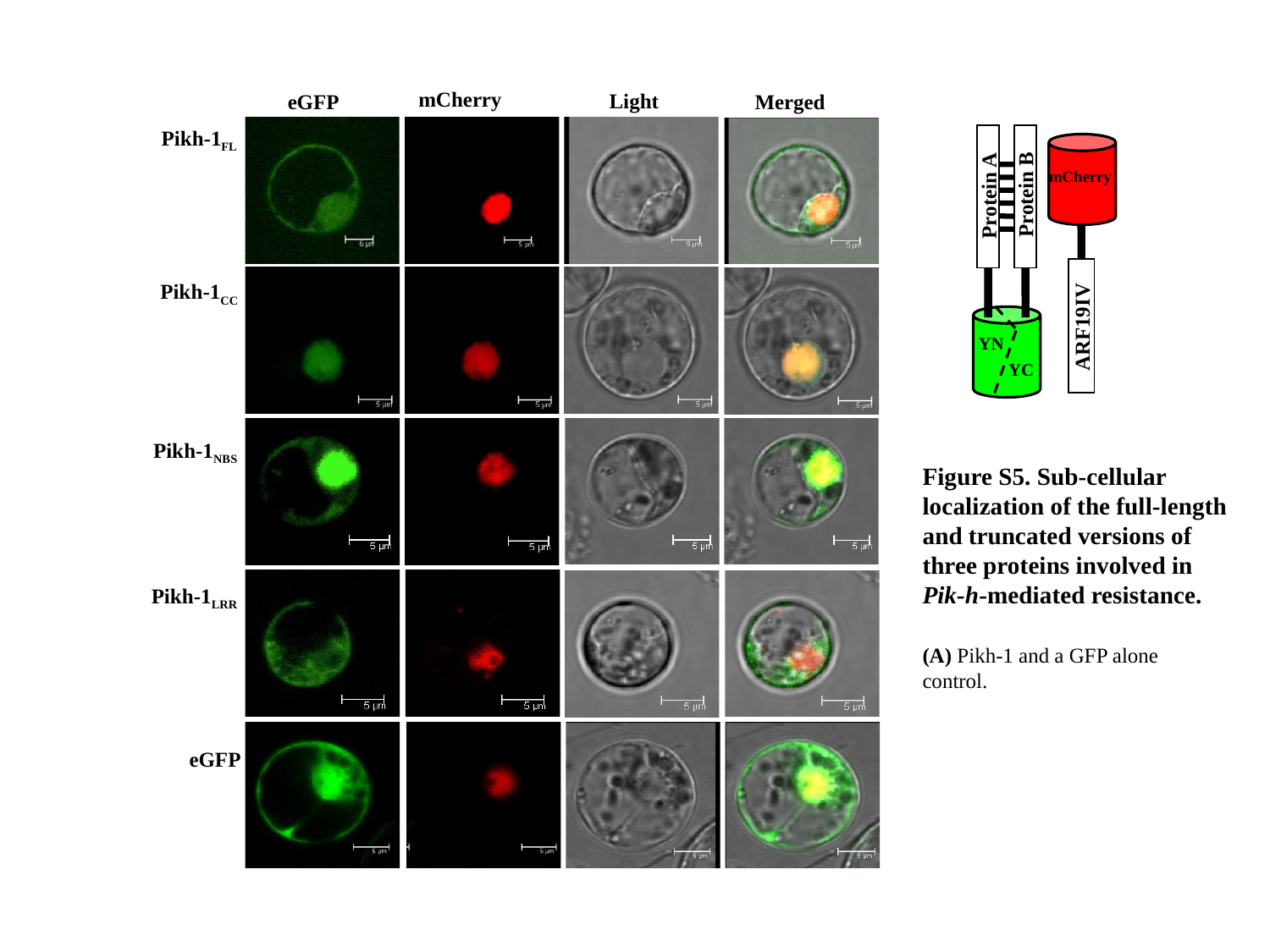

mCherry
Light
Merged
eGFP
Pikh-1FL
Pikh-1CC
Pikh-1NBS
Pikh-1LRR
eGFP
Protein B
Protein A
YN
YC
ARF19IV
mCherry
Figure S5. Sub-cellular localization of the full-length and truncated versions of three proteins involved in Pik-h-mediated resistance.
(A) Pikh-1 and a GFP alone control.

## Slide 2
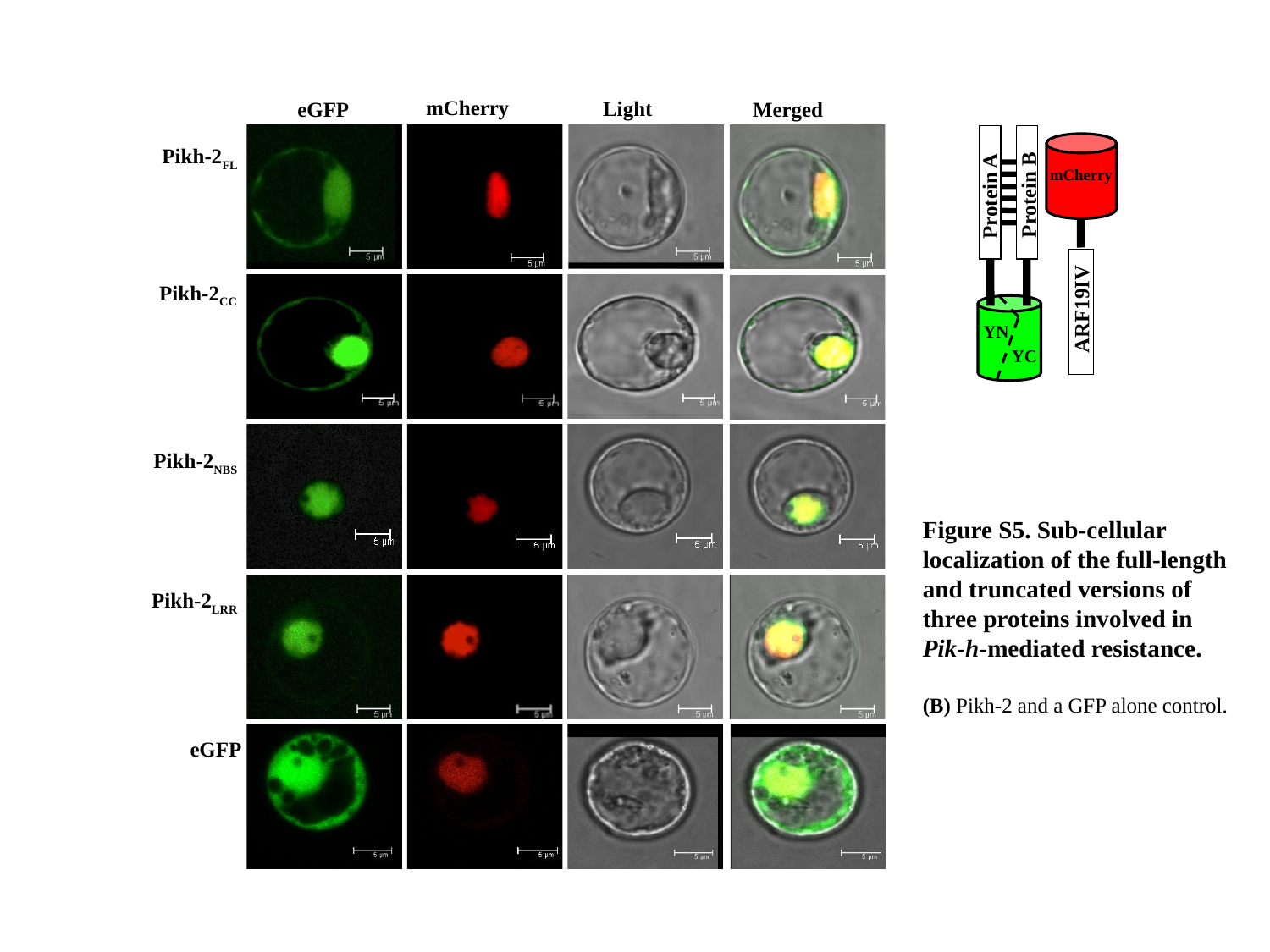

mCherry
Light
Merged
eGFP
Pikh-2FL
Pikh-2CC
Pikh-2NBS
Pikh-2LRR
eGFP
YN
YC
Protein B
Protein A
ARF19IV
mCherry
Figure S5. Sub-cellular localization of the full-length and truncated versions of three proteins involved in Pik-h-mediated resistance.
(B) Pikh-2 and a GFP alone control.

## Slide 3
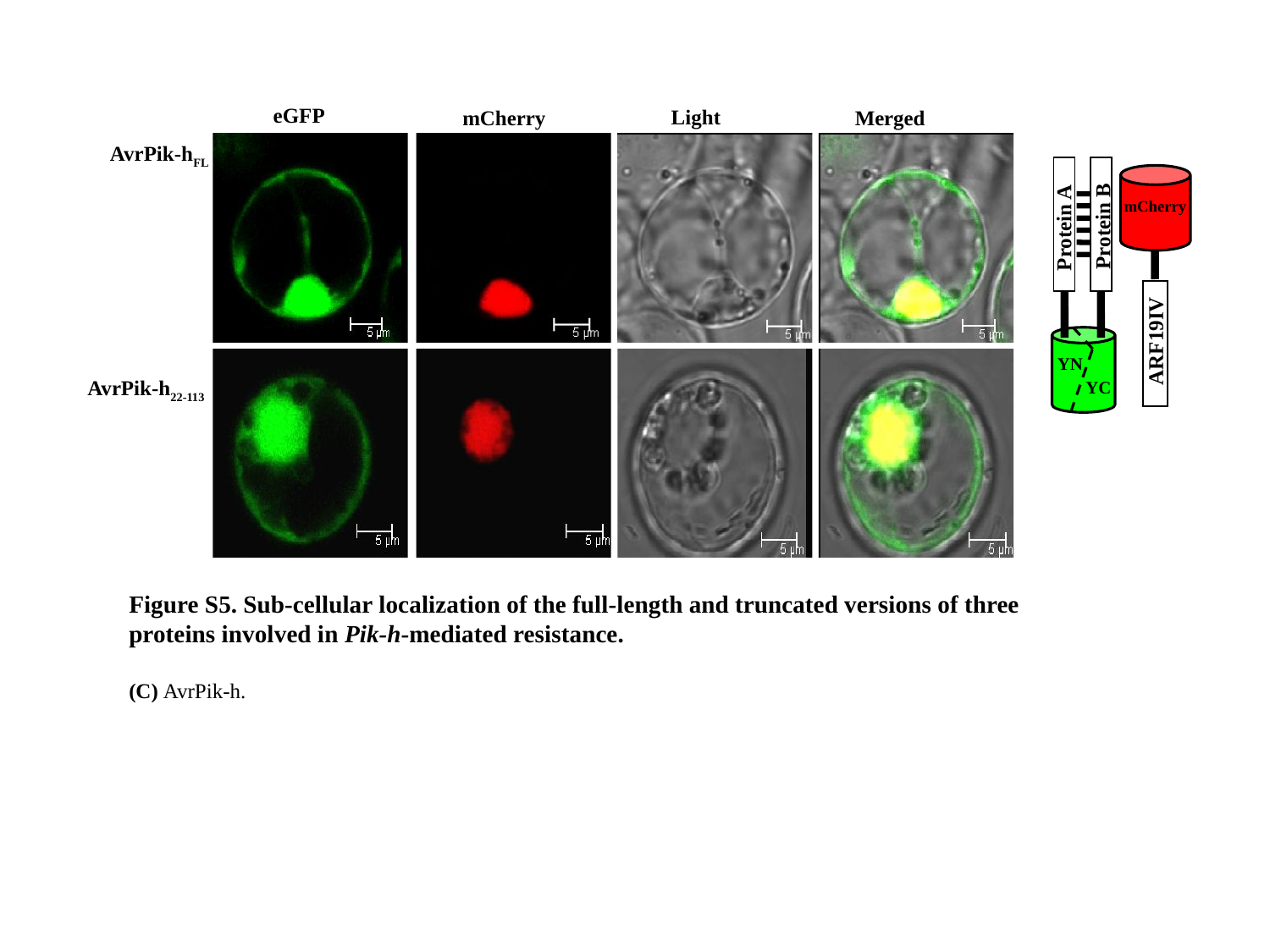

eGFP
Light
mCherry
Merged
AvrPik-hFL
AvrPik-h22-113
YN
YC
Protein B
Protein A
ARF19IV
mCherry
Figure S5. Sub-cellular localization of the full-length and truncated versions of three proteins involved in Pik-h-mediated resistance.
(C) AvrPik-h.

## Slide 4
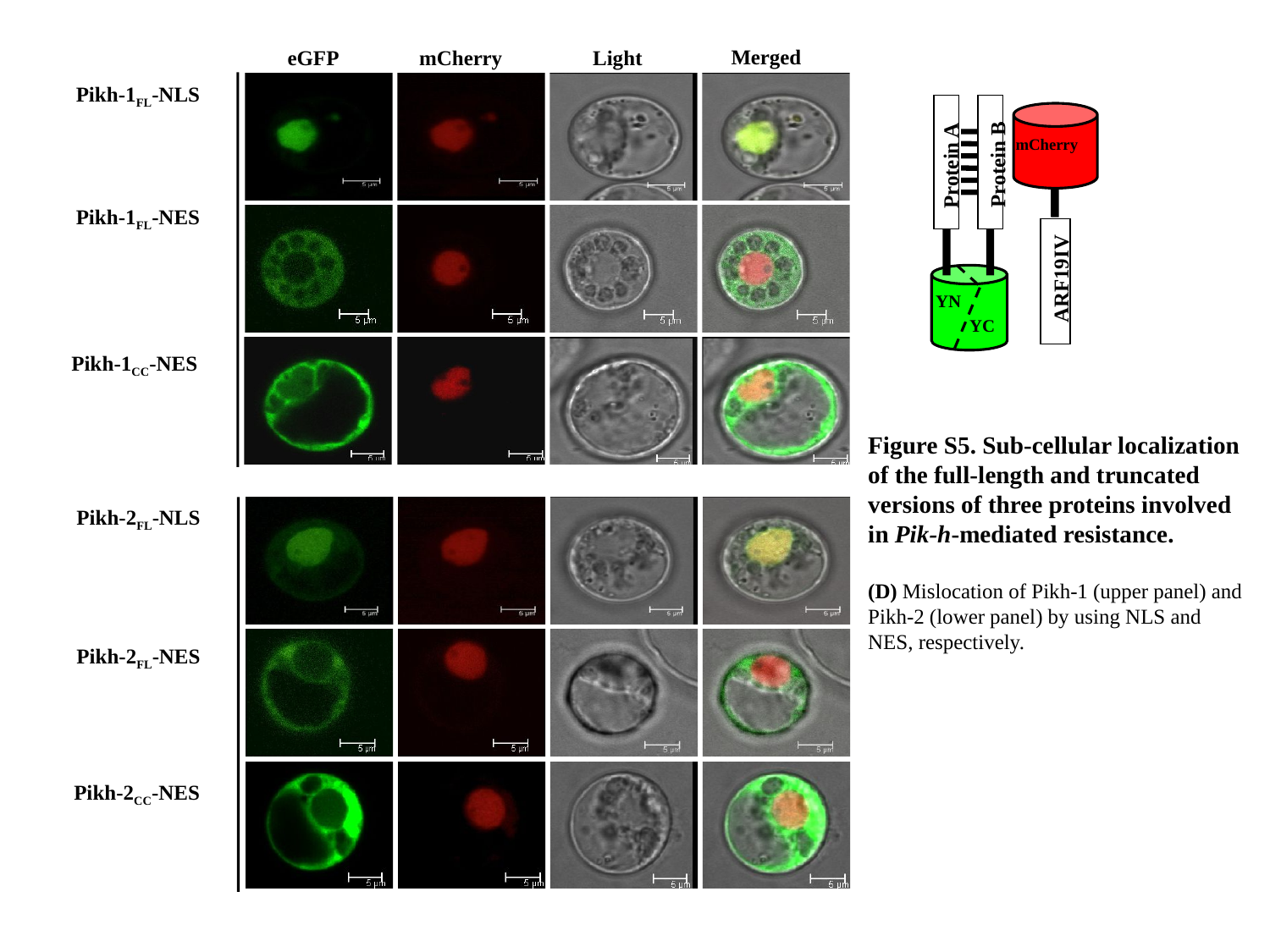

Merged
mCherry
Light
eGFP
Pikh-1FL-NLS
Pikh-1FL-NES
Pikh-1CC-NES
Pikh-2FL-NLS
Pikh-2FL-NES
Pikh-2CC-NES
YN
YC
Protein B
Protein A
ARF19IV
mCherry
Figure S5. Sub-cellular localization of the full-length and truncated versions of three proteins involved in Pik-h-mediated resistance.
(D) Mislocation of Pikh-1 (upper panel) and Pikh-2 (lower panel) by using NLS and NES, respectively.
