# Supplementary material for: Function and Interaction of the Coupled Genes Responsible for Pik-h Encoded Rice Blast Resistance
Source: PLoS One. 2014 Jun 4;9(6):e98067. doi: 10.1371/journal.pone.0098067 (PMC4045721; doi:10.1371/journal.pone.0098067)
Supplement: Table S1 — Primers, markers and constructs used in the study. (PDF) [file pone.0098067.s010.pdf]

Table S1. Primers, markers and constructs used in the study

Primer name	Forward primer	Enzyme site	Reverse primer	Enzyme site	Use of primers	Resultant construct
					**Gene cloning**	
KP3	TTTTGGCGCGCCGCCAGTGTCCACCAACCACAGTAATAAA	*Asc*I	TTTTGGCGCGCCGAACAGCCTGAGGAAGCAGAACATCGTC	*Asc*I	*Pikh-1* full-length gene	
KP3 ORF	TTTTGGCGCGCCGAGTAGCACTCAACGCAAAAGGGCATCGGC	*Asc*I	TTTTGGCGCGCCGAGCGGGGACTTAGAGTATTACATGAAGGA	*Asc*I	*Pihk-1* ORF	
KP4	TTTTGGCGCGCCGCAAGATCAGTACCATCACGAGTAATAGCA	*Asc*I	TTTTGGCGCGCCAGGGACAGAATGACAGTGTAAGTGAGTTTG	*Asc*I	*Pikh-2* full-length gene	
KP4 ORF	TTTGGCGCGCCAGCTGAAAGTTGCAGTGAGAAGTACAGA	*Asc*I	TTTGGCGCGCCTCAGATGATTGGTAAGTTCTCCGATTTG	*Asc*I	*Pikh-2* ORF	
					**RNAi analysis**	
KP3i1	TTTACTAGTGGTACCTGGCCAGATCTCCCAATCGT	*Spe*I-*Kpn*I	TTTGAGCTCGGATCCTGTGCCTGTGCAGGCTCAGT	*Sac*I-*Bam*HI	*Pikh-1* RNAi	
KP4i	CACCTTGAGCTGTCCGGCAAGTT		CAGTGACGATGCCATCAACAA		*Pikh-2* RNAi	
					**RACE**	
KP3-5RACE1	CGGCATCATCCAGGTCGTAGGACAGC				*Pikh-1* 5’ RACE	
KP3-5RACE2	CCATCCTTGCACGCCGCATCGTCATC				*Pikh-1* 5’ RACE nested primer	
KP3-3RACE1	CCATCGGCATCACCAACCTGAAGAGC				*Pikh-1* 3’ RACE	
KP3-3RACE2	TGAAGAAAGAAGCCGAGGAGCATCCC				*Pikh-1* 3’ RACE nested primer	
KP4-5RACE1	CGATCTGCGATCTGTTGTGCCCGTAC				*Pikh-2* 5’ RACE	
KP4-5RACE2	GAATCCTGAGGGAGGCGGTGGGCAAA				*Pikh-2* 5’ RACE nested primer	
KP4-3 RACE1	TCGCCCTTGAGCTGTCTGGCAAGTTGGA				*Pikh-2* 3’ RACE	
KP4-3 RACE2	AGCTACCCAAGTGGATCACCAGCATCAC				*Pikh-2* 3’ RACE nested primer	
					**Expression analysis**	
RRT5	GAAGCTCTGATCAACGGTATTCC		TCTTTGATCATCTTCGGGATACG		*Pikh-1*	
RRT17	TGAACTTCCACGATTGGATCCAC		ACATGGTTCTTGAATACATCATGTC		*Pikh-2*	
Actin	GAGACATTCAGCGTTCCAGC		GTATGTCGTCTCGTGGATGC		Internal reference	
PBZ1	GCCGCAAGTCATGTCCTAAAG		GCCGTCCTCGAGCTCGTA		Internal reference	
					**Transgenic plant test**	
Gus-linker	CATGAAGATGCGGACTTACG		ATCCACGCCGTATTCGG			
Hpt	CCGCGCGGATCCGGGTGCCTAATGAGTGAGC		TCCGGATGCCTCCGCTCGAAGTA			
					**Yeast two-hybrid assay**	
Y2H-kh1	TTGGCGCGCCATGGAGGCGGCTGCCATGGCCGT	*AscI*	TTGGCGCGCCGCTAGTAGTTTCTGTTTGAATTTCA	*AscI*	*Pikh-1* CDS	BD/ AD-kh1
Y2H-kh2	TTGGCGCGCCATGGAGTTGGTGGTAGGTGCTTC	*AscI*	TTGGCGCGCCTGCAGTGACGATGCCATCAACAAAT	*AscI*	*Pikh-2* CDS	BD/AD-kh2
Y2H-kh1$_{CC}$	TTGGCGCGCCATGGAGGCGGCTGCCATGGCCGT	*AscI*	TTGGCGCGCCCTTCACATCTTTCTTTGCTTGGCTG	*AscI*	*Pikh-1* CC domain	BD/AD-kh1$_{CC}$
Y2H-kh1$_{NBS}$	TTGGCGCGCCGAGACGACGCCGATGCTTGC	*AscI*	TTGGCGCGCCTGGGGAGGATGCACTAGTACT	*AscI*	*Pikh-1* NBS domain	BD/AD-kh1$_{NBS}$
Y2H-kh1$_{LRR}$	TTGGCGCGCCAGGTTGATTCGCCGGCTGTCTC	*AscI*	TTGGCGCGCCGGCGCTGATGCCAGGGGCGTCGC	*AscI*	*Pikh-1* LRR domain	BD/AD-kh1$_{LRR}$
Y2H-kh1$_{NBLRR}$	TTGGCGCGCCGAGACGACGCCGATGCTTGC	*AscI*	TTGGCGCGCCGCTAGTAGTTTCTGTTTGAATTTCA	*AscI*	*Pikh-1* NBLRR domain	BD/AD-kh1$_{NBSLRR}$
Y2H-k1$_{RATX1}$	TTGGCGCGCCATGAAAATCGTGTTCAAGATTCCCA	*AscI*	TT GGCGCGCCTCAATCTTCCTTTACTTGGCTGAC	*AscI*	*Pik-1* RATX1 domain	BD/ AD-k1 $_{RATX1}$
Y2H-Ks1$_{RATX1}$	TTGGCGCGCCATGAAAATCGTGTTCAAGATTCCCA	*AscI*	TTGGCGCGCCTCAATCTTCCTTTACTTGGCTGAC	*AscI*	*Piks-1* RATX1 domain	BD/ AD-ks1 $_{RATX1}$
Y2H-km1$_{RATX1}$	TTGGCGCGCCATGAAAATCGTGTTCAAGATTCCCA	*AscI*	TTGGCGCGCCTCAATCTTCCTTTGCTTGGCTGA	*AscI*	*Pikm-1* RATX1domain	BD/ AD-km1$_{RATX1}$
Y2H-kp1$_{RATX1}$	TTGGCGCGCCATGAAAATCGTGATCAAGGTTGCC	*AscI*	TTGGCGCGCCTCAATCTTTATTTGCTTGGCTGACC	*AscI*	*Pikp-1* RATX1 domain	BD/ AD-kp1$_{RATX1}$
Y2H-kh1$_{RATX1}$	TTGGCGCGCCATGAAAATCGTGATCAAGGTTGCC	*AscI*	TTGGCGCGCCTCAATCTTTCTTTGCTTGGCTGAC	*AscI*	*Pikh-1* RATX1 domain	BD/ AD-kh1$_{RATX1}$
Y2H-kh2$_{CC}$	TTGGCGCGCCATGGAGTTGGTGGTAGGTGCTTC	*AscI*	TTGGCGCGCCCCCCACAGGCTCCTTTATACCAATG	*AscI*	*Pikh-2* CC domain	BD/AD-kh2$_{CC}$
Y2H-kh2$_{NBS}$	TTGGCGCGCCATGAAGACGGTCATGGAGGAGC	*AscI*	TTGGCGCGCCGCCTTTTGTTGAACTTCCACGATT	*AscI*	*Pikh-2* NBS domain	BD/AD-kh2$_{NBS}$
Y2H-kh2$_{LRR}$	TTGGCGCGCCCTGAACTTGGCTCAAGTGAGATCAC	*AscI*	TTGGCGCGCCTGCAGTGACGATGCCATCAACAAAT	*AscI*	*Pikh-2* LRR domain	BD /AD-kh2$_{LRR}$
Y2H-kh2$_{NBLRR}$	TTCCCCCGGGTATGAAGACGGTCATGGAGGAGC	*AscI*	TTGGCGCGCCTGCAGTGACGATGCCATCAACAAAT	*AscI*	*Pikh-2* NBLRR domain	BD/AD-kh2$_{NBSLRR}$
Y2H-Ak	TTGGCGCGCCATGCGTGTTACCACTTTTA	*AscI*	TTGGCGCGCCAAAGCCGGGCCTTTTTTTCC	*AscI*	*AvrPik-h* CDS	BD/AD-Ak
Y2H-Ak$_{22-113}$	TTGGCGCGCCGAAACGGGCAACAAATA	*AscI*	TTGGCGCGCCAAAGCCGGGCCTTTTTTTCC	*AscI*	*AvrPik-h* truncated CDS	BD/AD-Ak$_{22-113}$

Y2H-Ak$_{53-113}$	TTGGCGCGCCGAATGTTTTTGGTTTATG	*AscI*	TTGGCGCGCCAAAGCCGGGCCTTTTTTTCC	*AscI*	*AvrPik-h* truncated CDS	BD/AD-Ak$_{53-113}$
Y2H-Ak$_{1-107}$	TTGGCGCGCCATGCGTGTTACCACTTTTA	*AscI*	TTGGCGCGCCCCCCAAAACAATCCAACCTG	*AscI*	*AvrPik-h* truncated CDS	BD/AD-Ak$_{1-107}$
					**Pull-down assay**	
Pd-kh1	TTGGCGCGCCATGGAGGCGGCTGCCATGGCCGT	*AscI*	TTGGCGCGCCGCTAGTAGTTTCTGTTTGAATTTCA	*AscI*	*Pikh-1* CDS	kh1-Flag
Pd-kh1$_{CC}$	TTGGCGCGCCATGGAGGCGGCTGCCATGGCCGT	*AscI*	TTGGCGCGCCCTTCACATCTTTCTTTGCTTGGCTG	*AscI*	*Pikh-1* CC domain	kh1$_{CC}$-Flag
Pd-kh2$_{CC}$	TTGGCGCGCCATGGAGTTGGTGGTAGGTGCTTC	*AscI*	TTGGCGCGCCTCACCCCACAGGCTCCTTTATACCAATG	*AscI*	*Pikh-2* CC domain	Kh2$_{CC}$-GST
Pd-Ak	TTGGCGCGCCATGCGTGTTACCACTTTTA	*AscI*	TTGGCGCGCCTTAAAAGCCGGGCCTTTTTTTCC	*AscI*	*AvrPik-h* CDS	Ak-GST
Pd-Ak$_{22-113}$	TTGGCGCGCCATGGAAACGGGCAACAAATA	*AscI*	TTGGCGCGCCTTAAAAGCCGGGCCTTTTTTTCC	*AscI*	*AvrPik-h* truncated CDS	Ak$_{22-113}$-GST
					**BiFC analysis**	
BiFC-kh1	TTGGCGCGCCATGGAGGCGGCTGCCATGGCCGT	*AscI*	TTGGCGCGCC T GCTAGTAGTTTCTGTTTGAATTTCA	*AscI*	*Pikh-1* CDS	YCE-kh1
BiFC-kh1$_{CC}$	TTGGCGCGCCATGGAGGCGGCTGCCATGGCCGT	*AscI*	TTGGCGCGCC T CTTCACATCTTTCTTTGCTTGGCTG	*AscI*	*Pikh-1* CC domain	YCE-kh1$_{CC}$
BiFC-kh2$_{CC}$	TTGGCGCGCCATGGAGTTGGTGGTAGGTGCTTC	*AscI*	TTGGCGCGCC T CCCCACAGGCTCCTTTATACCAATG	*AscI*	*Pikh-2* CC domain	YNE-kh2$_{CC}$
BiFC-Ak	TTGGCGCGCCATGCGTGTTACCACTTTTA	*AscI*	TTGGCGCGCC T TTAAAAGCCGGGCCTTTTTTTCC	*AscI*	*AvrPik-h* CDS	YNE-Ak
BiFC-Ak$_{22-113}$	TTGGCGCGCCATGGAAACGGGCAACAAATA	*AscI*	TTGGCGCGCC T TTAAAAGCCGGGCCTTTTTTTCC	*AscI*	*AvrPik-h* truncated CDS	YNE-Ak$_{22-113}$
NLS-*Xba I*	AATCTAGAATGCCT AAA AAG AAG CGT AAG GT	*Xba I*	AATCTAGA AGAATTCACGGATCCAGGGTC	*Xba I*	NLS	
NES-*Xba I*	ATTTCTAGAATGAATGAATTAGCCTTGAAA	*Xba I*	ATTTCTAGATGTCTTGTTGATATCAAGA	*Xba I*	NES	
					**Subcellular localization analysis**	
eGFP-kh1	TTGGCGCGCCATGGAGGCGGCTGCCATGGCCGT	*AscI*	TTGGCGCGCC T GCTAGTAGTTTCTGTTTGAATTTCA	*AscI*	*Pikh-1* CDS	Pikh-1-eGFP
eGFP-kh1$_{CC}$	TTGGCGCGCCATGGAGGCGGCTGCCATGGCCGT	*AscI*	TTGGCGCGCC T CTTCACATCTTTCTTTGCTTGGCTG	*AscI*	*Pikh-1* CC domain	Pikh-1$_{CC}$-eGFP
eGFP-kh1$_{NBS}$	TTGGCGCGCCGAGACGACGCCGATGCTTGC	*AscI*	TTGGCGCGCC T TGGGGAGGATGCACTAGTACT	*AscI*	*Pikh-1* NBS domain	Pikh-1$_{NBS}$-eGFP
eGFP-kh1$_{LRR}$	TTGGCGCGCCAGGTTGATTCGCCGGCTGTCTC	*AscI*	TTGGCGCGCC T GGCGCTGATGCCAGGGGCGTCGC	*AscI*	*Pikh-1* LRR domain	Pikh-1$_{LRR}$-eGFP
eGFP-kh2	TTGGCGCGCCATGGAGTTGGTGGTAGGTGCTTC	*AscI*	TTGGCGCGCC T TGCAGTGACGATGCCATCAACAAAT	*AscI*	*Pikh-2* CDS	Pikh-2-eGFP
eGFP-kh2$_{CC}$	TTGGCGCGCCATGGAGTTGGTGGTAGGTGCTTC	*AscI*	TTGGCGCGCC T CCCCACAGGCTCCTTTATACCAATG	*AscI*	*Pikh-2* CC domain	Pikh-2$_{CC}$-eGFP
eGFP-kh2$_{NBS}$	TTGGCGCGCCATGAAGACGGTCATGGAGGAGC	*AscI*	TTGGCGCGCC T GCCTTTTGTTGAACTTCCACGATT	*AscI*	*Pikh-2* NBS domain	Pikh-2$_{NBS}$-eGFP
eGFP-kh2$_{LRR}$	TTGGCGCGCCCTGAACTTGGCTCAAGTGAGATCAC	*AscI*	TTGGCGCGCC T TGCAGTGACGATGCCATCAACAAAT	*AscI*	*Pikh-2* LRR domain	Pikh-2$_{LRR}$-eGFP
NLS- *NotI*	AAGCGGCCGCATGCCT AAA AAG AAG CGT AAG GT	*NotI*	AAGCGGCCGCAGAATTCACGGATCCAGGGTC	*NotI*	NLS	
NES- *NotI*	ATTGCGGCCGCATGAATGAATTAGCCTTGAAA	*NotI*	ATTGCGGCCGCCTGTCTTGTTGATATCAAGA	*NotI*	NES	
					**Transient overexpression in tabacoo**	
Te-kh1	TTGGCGCGCCATGGAGGCGGCTGCCATGGCCGT	*AscI*	TTGGCGCGCCTCAGCTAGTAGTTTCTGTTTGAATTTCA	*AscI*	*Pikh-1* CDS	35S-kh1
Te-kh2	TTGGCGCGCCATGTATGGAGTTGGTGGTAGGTGCTTC	*AscI*	TTGGCGCGCCTCATTGCAGTGACGATGCCATCAACAAAT	*AscI*	*Pikh-2* CDS	35S-kh2
Te-Ak	TTGGCGCGCCATGCGTGTTACCACTTTTA	*AscI*	TTGGCGCGCCTTAAAAGCCGGGCCTTTTTTTCC	*AscI*	*AvrPik-h* CDS	35S-Ak
Te-Ak$_{22-113}$	TTGGCGCGCCATGGAAACGGGCAACAAATA	*AscI*	TTGGCGCGCCTTAAAAGCCGGGCCTTTTTTTCC	*AscI*	*AvrPik-h* truncated CDS	35S-Ak$_{22-113}$
Te-kh2$_{CC}$	TTGGCGCGCCATGGAGTTGGTGGTAGGTGCTTC	*AscI*	TTGGCGCGCCTCACCCCACAGGCTCCTTTATACCAATG	*AscI*	*Pikh-2* CC domain	35S-kh2$_{CC}$
Te-kh2$_{NBS}$	TTGGCGCGCCATGAAGACGGTCATGGAGGAGC	*AscI*	TTGGCGCGCCTCAGCCTTTTGTTGAACTTCCACGATT	*AscI*	*Pikh-2* NBS domain	35S-kh2$_{NBS}$
Te-kh2$_{LRR}$	TTGGCGCGCCATGCTGAACTTGGCTCAAGTGAGATCAC	*AscI*	TTGGCGCGCCTCATGCAGTGACGATGCCATCAACAAAT	*AscI*	*Pikh-2* LRR domain	35S-kh2$_{LRR}$
Te-kh2$_{NBSLRR}$	TTCCCCCGGGATGTATGAAGACGGTCATGGAGGAGC	*AscI*	TTGGCGCGCCTCATGCAGTGACGATGCCATCAACAAAT	*AscI*	*Pikh-2* NBLRR domain	35S-kh2$_{NBSLRR}$
NLS-T	TATGCCT AAA AAG AAG CGT AAG GT	*Xcm I*	ATTGGCGCGCCATGCCT AAA AAG AAG CGT AAG GT	*AscI*	NLS	35S-kh2-NLS
NES-T	TATGAATGAATTAGCCTTGAAA	*Xcm I*	ATTGGCGCGCCCTGTCTTGTTGATATCAAGA	*AscI*	NES	35S-kh2-NES
kh2-prom	CGCGGATCCCGATGCCCTTTTGCGTTGAGTG	*BamHI*	CGCGGATCC GGCGCGCC CTTGTTCTCTGTACTTCTCACTGC	*BamHI*	*Pikh-2* promoter	
HA-kh2	AACCCGGGATGTACCCATACGATGTTCCAGATT	*Sma I*	AACCCGGGTGCAGTGACGATGCCATCAACAAAT	*Sma I*	HA-*Pikh-2*	NP-kh2
					**Transient expression in rice protoplast**	
kh1-prom	CGCGGATCCCTTGTTCTCTGTACTTCTCACTGC	*BamHI*	CGCGGATCC GGCGCGCC CGATGCCCTTTTGCGTTGAGTG	*BamHI*	*Pikh-1* promoter	
kh2-prom	CGCGGATCCCGATGCCCTTTTGCGTTGAGTG	*BamHI*	CGCGGATCC GGCGCGCC CTTGTTCTCTGTACTTCTCACTGC	*BamHI*	*Pikh-2* promoter	
Luc-kh1	TTGGCGCGCCATGGAGGCGGCTGCCATGGCCGT	*AscI*	TTGGCGCGCCTCAGCTAGTAGTTTCTGTTTGAATTTCA	*AscI*	*Pikh-1* CDS	Luc-kh1
Luc-kh1$_{CC}$	TTGGCGCGCCATGGAGGCGGCTGCCATGGCCGT	*AscI*	TTGGCGCGCCTCACTTCACATCTTTCTTTGCTTGGCTG	*AscI*	*Pikh-1* CC domain	Luc-kh1$_{CC}$

Luc-kh1$_{NBS}$	TTGGCGCGCCATGGAGACGACGCCGATGCTTGC	*AscI*	TTGGCGCGCCTCATGGGGAGGATGCACTAGTACT	*AscI*	*Pikh-1* NBS domain	Luc- kh1$_{NBS}$
Luc-kh1$_{LRR}$	TTGGCGCGCCATGAGGTTGATTCGCCGGCTGTCTC	*AscI*	TTGGCGCGCCTCAGGCGCTGATGCCAGGGGCGTCGC	*AscI*	*Pikh-1 LRR domain*	Luc-kh1$_{LRR}$
Luc-kh2	TTGGCGCGCCATGTATGGAGTTGGTGGTAGGTGCTTC	*AscI*	TTGGCGCGCCTCATTGCAGTGACGATGCCATCAACAAAT	*AscI*	*Pikh-2* CDS	Luc-kh2
Luc-kh2$_{CC}$	TTGGCGCGCCATGGAGTTGGTGGTAGGTGCTTC	*AscI*	TTGGCGCGCCTCACCCCACAGGCTCCTTTATACCAATG	*AscI*	*Pikh-2* CC domain	Luc-kh2$_{CC}$
Luc-kh2$_{NBS}$	TTGGCGCGCCATGAAGACGGTCATGGAGGAGC	*AscI*	TTGGCGCGCCTCAGCCTTTTGTTGAACTTCCACGATT	*AscI*	*Pikh-2* NBS domain	Luc-kh2$_{NBS}$
Luc-kh2$_{LRR}$	TTGGCGCGCCATGCTGAACTTGGCTCAAGTGAGATCAC	*AscI*	TTGGCGCGCCTCATGCAGTGACGATGCCATCAACAAAT	*AscI*	*Pikh-2* LRR domain	Luc-kh2$_{LRR}$
Luc-Ak	TTGGCGCGCCATGCGTGTTACCACTTTTA	*AscI*	TTGGCGCGCCTTAAAAGCCGGGCCTTTTTTTCC	*AscI*	*AvrPik-h* CDS	Ubi- Ak
Luc-Ak$_{22-113}$	TTGGCGCGCCGAAACGGGCAACAAATA	*AscI*	TTGGCGCGCCTTAAAAGCCGGGCCTTTTTTTCC	*AscI*	*AvrPik-h* truncated CDS	Ubi- Ak$_{22-113}$
FLAG-Nos	AACCCGGGATGGATTACAAGGATGATGATGATA	*Sma I*	AACCCGGG CGATCTAGTAACATAGATGA	*Sma I*	FLAG-tagged fragments	
HA-Nos	AACCCGGG ATGTACCCATACGATGTTCCAGATT	*Sma I*	AACCCGGG CGATCTAGTAACATAGATGA	*Sma I*	HA-tagged fragments	
